# Textures and traction: how tube-dwelling polychaetes get a leg up

**DOI:** 10.1111/ivb.12079

**Published:** 2015-03-03

**Authors:** Rachel Ann Merz

**Affiliations:** Department of Biology, Swarthmore CollegeSwarthmore, Pennsylvania, 19081, USA

**Keywords:** Annelida, attachment, chaetae, paleae, uncini

## Abstract

By controlling the traction between its body and the tube wall, a tube-dwelling polychaete can move efficiently from one end of its tube to the other, brace its body during normal functions (e.g., ventilation and feeding), and anchor within its tube avoiding removal by predators. To examine the potential physical interaction between worms and the tubes they live in, scanning electron microscopy was used to reveal and quantify the morphology of worm bodies and the tubes they produce for species representing 13 families of tube-dwelling polychaetes. In the tubes of most species there were macroscopic or nearly macroscopic (∼10 μm–1 mm) bumps or ridges that protruded slightly into the lumen of the tube; these could provide purchase as a worm moves or anchors. At this scale (∼10 μm-1 mm), the surfaces of the chaetal heads that interact with the tube wall were typically small enough to fit within spaces between these bumps (created by the inward projection of exogenous materials incorporated into the tube wall) or ridges (made by secretions on the interior surface of the tube). At a finer scale (0.01–10 μm), there was a second overlap in size, usually between the dentition on the surfaces of chaetae that interact with the tube walls and the texture provided by the secreted strands or microscopic inclusions of the inner linings. These linings had a surprising diversity of micro-textures. The most common micro-texture was a “fabric” of secreted threads, but there were also orderly micro-ridges, wrinkles, and rugose surfaces provided by microorganisms incorporated into the inner tube lining. Understanding the fine structures of tubes in conjunction with the morphologies of the worms that build them gives insight into how tubes are constructed and how worms live within them.

The tubes of polychaete annelids have long been of interest to a variety of scientists. Paleontologists have examined them for insight into the early biosphere and the evolution of the annelids (Gringras et al. 2002; Schweitzer et al. 2005; Chen et al. 2008). Ecologists have recognized the role tubes play in stabilizing sediments and providing microhabitats that enrich and modify marine habitats (Fager 1964; Multer & Milliman 1967; Woodin 1974; Callaway et al. 2010; Ryer et al. 2013). Invertebrate biologists have studied the mechanisms by which tubes are built or secreted (Watson 1903, 1928; Nicol 1930; Fitzsimons 1965; Defretin 1971; Gaill & Hunt 1988). Recently, materials scientists have been interested in their properties with an eye towards the construction of new biomimetic materials for human use (Stewart et al. 2004; Zhao et al. 2005; Sun et al. 2007; Shao et al. 2009; Shah et al. 2014). Despite these multifaceted approaches, rather little attention has been focused on how the resident worms live in these structures or what features might exist that would allow worms to take secure refuge within tubes, brace their bodies during normal functions (e.g., ventilation and feeding), or to move efficiently from one end of a tube to the other (Merz & Woodin 2006).

Are there common features associated with the linings of tubes that allow worms to achieve these functions? If so, how do they relate to the morphology of the worms? To examine these questions I have surveyed more than 16 polychaete species representing 13 families sampled broadly from tube-dwelling lineages. My goal is to present a sample of the textures of the inner linings of tubes, to gain insight into how the tubes are produced, and to compare the size ranges of worm and tube morphologies. This comparison forms the basis for understanding how these worms might interact with their tubes. Examining the morphological patterns across a wide variety of species makes it possible to recognize similar solutions to the mechanical problems presented by residing and moving within tubes and to identify which lineages have unusual characteristics.

## Overview of polychaete tube composition and construction

Polychaete tubes are strong, resilient structures that support and protect their residents under a variety of challenging mechanical settings. Tubes withstand high-energy intertidal waves (Multer & Milliman 1967; Stewart et al. 2004), provide effective barriers that moderate the thermal and chemical extremes associated with deep-sea hydrothermal vents (Zbinden et al. 2003), act as cantilevers supporting feeding tentacles into moving water (Merz 1984), and give protection from predators (Dill & Fraser 1997; Kicklighter & Hay 2007). In addition, many tubes persist for months if not longer after the worm is gone (Fitzsimons 1965; Le Cam et al. 2011).

Polychaete tubes are stratified composite structures. The outer layer often incorporates exogenous materials (sediment, shell fragments, algae, etc.). The inner sheath is usually comprised of successive layers made of sheets (Shillito et al. 1995; Ravaux et al. 2000) or fine threads (Daly 1973; Nishi 1993) that are produced after the outer layer is formed. Tube walls are thickened as these new inner layers are subsequently laid down by the worm.

Typically, a settling larva of a tube-dwelling polychaete makes the initial tube (e.g., Carpizo-Ituarte & Hadfield 1998; Pernet 2001). Depending on species, this original tube may be produced in minutes (Carpizo-Ituarte & Hadfield 1998) or hours (Pinedo et al. 2000) by secretions from the surface of the body. For species that reside in the same tube for long periods (in many cases the life-span of the resident), tubes are elongated by the application of new tube material to the existing rim. The method by which this is done varies with species but typically involves specialized glands associated with the anterior segments of the worm. In some sabellids, particles captured on the feeding tentacles are incorporated with glandular secretions, and this mixture is applied as a coil to the edge of the tube as the worm turns on its long axis (Nicol 1930; Fitzsimons 1965; Defretin 1971). Pectinarids, sabellarids, owenids, terebellids, and spionids, like masons, select sediment grains with their tentacles or palps and glue them to the pre-existing tube edge (Fager 1964; Defretin 1971; Pinedo et al. 2000; Zhao et al. 2005; Noffke et al. 2009; Fournier et al. 2010).

In contrast, there are other species that readily make entire new tubes throughout their lives (e.g., nereidids, maldanids, some sabellids; see Table1), and in these there may be no specific anterior tube constructing organs. Instead tube-secreting glands may exist along the body, in some cases associated with parapodia (Defretin 1971; Bonar 1972). For instance, *Platynereis dumerilii* (audouin & Milne Edwards 1834) has glandular masses on the parapodia that secrete successive layers of fine threads forming an elastic tubular coating in which algae or other materials may be incorporated (Daly 1973). In burrowing species, epidermal glands secrete material that forms the lining of the burrow and to which sediment adheres (Bonar 1972).

**Table 1:** Species illustrated in this paper and their source, habitat, tube orientation, and ability to regenerate tubes. If a worm is removed from its original tube its ability to regenerate a new tube varies with species. Based on observations in the laboratory, “readily regenerates” describes worms that can produce a new tube in a few minutes while “regenerates” refers to those that can produce a new tube in hours. If the worm remains indefinitely tubeless in a sea table even if building materials are available it is considered to be “unable to regenerate” (although these same species can often extend an artificial tube with native materials). The categories of “may regenerate” and “unlikely to regenerate tube” are based on the behavior of related taxa. Organization of families follows Weigert et al. (2014); feeding patterns from observation and Jumars et al. (2015).

Family Species Source	Collection habitat	Tube and worm orientation; feeding mode; ability to regenerate tube
Oweniidae		
*Owenia collaris*Hartman 1955 Charleston, OR (43.35133, −124.31491)	Muddy sand	Vertical with anterior tip extended above sediment-water interface; suspension feeder & surface deposit feeder; regenerates tube
Chaetopteridae		
*Mesochaetopterus taylori*Monro 1928 False Bay, WA (48.48267, −123.07299)	Muddy sand	Vertical, anterior tip elevated above sediment-water interface, posterior in sand; feeds from mucus net within tube; may be able to regenerate tube
Siboglinidae		
*Oasisia* sp. *SCRIPPS, DSV Alvin 4093* (−31.51869, −112.02638)	Deep sea vent 2235 m	Variable with anterior end extended freely in water, tube base attached to hard surface; nutrition presumably from symbiotic bacteria, osmotroph; unlikely to regenerate tube
Sabellaridae		
*Sabellaria cementarium*Moore 1906 Sunset Bay, OR (43.33085, −124.38167)	Rock rubble	Variable, attached to rock and other tubes; passive suspension feeder; unable to regenerate tube
*Idanthyrsus macropaleus* (Schmarda 1861) Sunset Bay, OR (43.33085, −124.38167)	Rock rubble	Variable, attached to rock and other tubes; passive suspension feeder; unable to regenerate tube
Serpulidae		
*Serpula columbiana*Johnson 1901 Charleston, OR (43.34600, −124.32798)	On mussel shell on pilings	Variable, attached to rock, shell and other hard surfaces; active/passive suspension feeder; unable to regenerate tube
Sabellidae		
*Schizobranchia insignis*Bush 1905 Charleston, OR (43.34600, −124.32798)	Pilings, floating dock	Usually vertical, anterior end extended freely in water, base attached to hard surface; active/passive suspension feeder; unable to regenerate tube
*Eudistylia vancouveri* (Kinberg 1866) Charleston, OR (43.34600, −124.32798)	Pilings, floating dock	Usually vertical, anterior end extended freely in water, base attached to hard surface; active/passive suspension feeder; unable to regenerate tube
Terebellidae		
*Pista brevibranchiata*Moore 1923 Charleston, OR (43.35133, −124.31491)	Soft sediment	Vertical, anterior end above sediment-water interface, posterior end in sediment; suspension feeder, detritivore; may be able to regenerate tube
Pectinariidae		
*Pectinaria gouldii* (Verrill 1874) Belmar, NJ (40.18639, −74.03028)	Muddy sand	Vertical, posterior end at sediment-water interface, anterior end buried in sand, subsurface deposit feeder; unable to regenerate tube
Ampharetidae		
*Schistocomus hiltoni*Chamberlin 1919 Sunset Bay, OR (43.33085, −124.38167)	Rock rubble	Variable, attached to rock and other hard surfaces; surface deposit feeder; may be able to regenerate tube
Alvinellidae		
*Alvinella pompejana*Desbruyères & *Laubier 1980* SCRIPPS, Acc. #A61 (20.85, −109.0)	Deep sea vent	Variable, attached to hard surfaces and other tubes; subsists on vent bacteria; unlikely to be able to regenerate tube
Maldanidae		
*Clymenella torquata* (Leidy 1855) Belmar, NJ (40.18639, −74.03028)	Muddy sand	Vertical with posterior end at sediment-water interface, subsurface deposit feeder; readily regenerates tube
Onuphidae		
*Diopatra ornata*Moore 1911 Charleston, OR (43.35133, −124.31491)	Muddy sand	Vertical, anterior end above sediment-water interface, posterior end in sand; herbivore, omnivorous, feeding from anterior end of tube; regenerates tube
Nereididae		
*Platynereis bicanaliculata* (Baird 1863) Sunset Bay, OR (43.33085, −124.38167)	Rock rubble	Variable, attached to algae and rock; omnivorous, consumes algae and diatoms; readily regenerates new tube

There are two functional categories of secretions associated with tube building—“glues” that attach exogenous materials to the tube, and “plastics” that are extruded and become load-bearing structures or linings. In both cases, the secretions are produced as liquids that harden in the presence of seawater (Bonar 1972; Simkiss & Wilber 1989; Stewart et al. 2004; Vinn et al. 2008). The “glues” in sabellarids, pectinarids, and terebellids have been analyzed (Stewart et al. 2004; Zhao et al. 2005; Fournier et al. 2010) and consist of proteins that contain significant levels of phosphate, calcium, and magnesium. The “plastics” come from a variety of glands that vary in position, morphology, and product (Gaill & Hunt 1988; Hausen 2005). Some of these glands secrete proteins, and others polysaccharides; analyses of tubes reflect those constituents. All tubes seem to contain protein (Gaill & Hunt 1988). For instance, the tubes of *Sabella* and *Spirographis* are made of 82–88% protein and a smaller amount of carbohydrate in the form of the polysaccharide hyaluronic acid (Defretin 1971). The tubes of the deep-sea species *Riftia pachyptila*Jones 1981 contain the polysaccharide β-chitin associated with protein (Shillito et al. 1995; Chamoy et al. 2001). In serpulids, some cirratulids, and one sabellid, the worms generate calcium carbonate tubes either by secreting a mixture of calcium granules in an acid mucopolysaccharide matrix, or by producing an organic matrix on which calcium is deposited (Fischer et al. 2000; Vinn et al. 2008). Therefore, polychaete tubes are often protein-rich, made of tough polysaccharides (e.g., β-chitin), and may have high mineral content (from biomineralized cements, calcium carbonate secretions, or from incorporated sediment), and as a result are strong and resistant to wear (Fitzsimons 1965; Le Cam et al. 2011).

## Methods

### Collection and preparation of specimens

To gain a broad phylogenetic perspective, I examined species from across the spectrum of polychaete taxa (Table1). The phylogeny of polychaetes has not yet been fully resolved (e.g., see Struck et al. 2007, 2011; Pleijel et al. 2009; Weigert et al. 2014), so I selected species guided by both a traditional morphology-based phylogeny (Rouse & Fauchald 1997) and by less inclusive but more recent molecular sequence-based analyses (Struck et al. 2007, 2011; Weigert et al. 2014). As a mode of checking the internal consistency of my techniques, I examined at least three individuals from each species (to confirm the presence of individual features) and twice chose closely related species (*Sabellaria cementarium* and *Idanthyrsus macropaleus* [family Sabellaridae] and *Schizobranchia insignis* and *Eudistylia vancouveri* [family Sabellidae]) to see if they would produce similar overall patterns. For issues having to do with practicality of viewing with scanning electron microscopy (SEM), all the individual worms examined had diameters from 0.5 to 5 mm.

Except for the two preserved species that were borrowed from Scripps Institution of Oceanography (*Alvinella pompejana* and *Oasisia* sp.), I collected live worms in their tubes by hand (for locations, tube orientation, feeding mode, and ability to regenerate tubes, see Table1). I carefully removed live worms from their tubes and then fixed both worms and tubes in 4% formalin in seawater for at least 48 h. The preserved specimens were then transferred through an ascending series of ethanol solutions (10, 20, 30, 50, 70, 85, 95, and 3X 100%, with 1 h at each step; specimens were incubated in the last change of 100% ethanol for at least 8 h). Dehydrated specimens were submerged in 100% hexamethyldisilazane (HMDS) for at least 8 h, a second bath of HMDS for at least 1 h, and then allowed to air dry (Nation 1983; Barré et al. 2006). Specimens were mounted on stubs using double-stick tape, adhesive carbon or copper tabs, or with silver paint, and then were sputter coated with gold-palladium and viewed either with a Zeiss Ultra-55 SEM at the University of Oregon or with a Philips XL 20 SEM at the University of Pennsylvania.

### Morphometrics

For comparative purposes, I measured features on the worms’ bodies and tubes that were shared among species and that might be related to locomotion and attachment within the tube. Measurements were made from SEM images. To diminish error, the same features were examined from different perspectives and magnifications.

**Figure 1:**
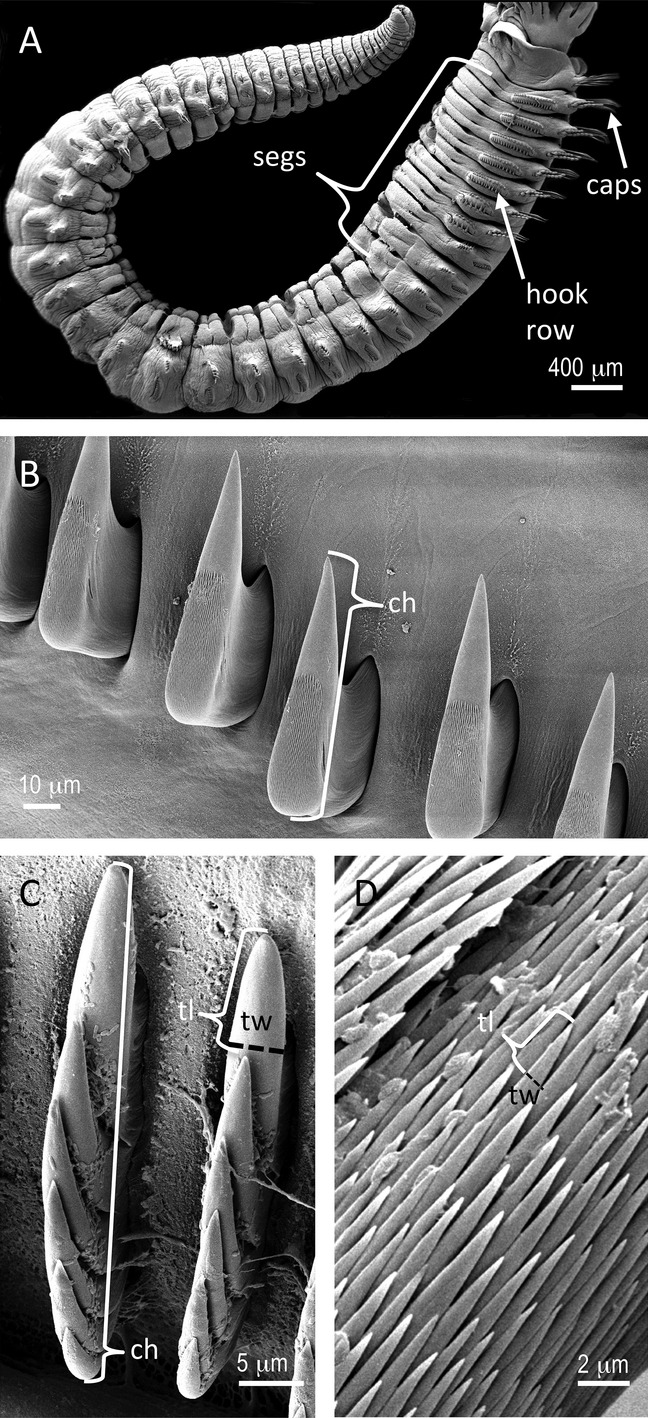
Morphological features of worm bodies that were measured for comparison with tube structures. A. The widest part of a tube-dwelling worm is most likely to interact with the tube wall. As an example, the bracket on this sabellid polychaete, *Schizobranchia insignis* (whole worm *sans* feeding crown) indicates the segments (segs) where measurements were taken. Capillary chaetae (caps) and a row of hooks or uncini occur on each segment in this species. B. The length measurement of the chaetal head (ch) of a thoracic uncinus from a hook row of one of the previously indicated segments. C. Multi-dentate thoracic uncini from the serpulid, *Serpula columbiana*. In white, the brackets indicated the length of the whole chaetal head (ch) and the tooth length (tl) of one of the micro-teeth. The black dashed line indicates that micro-tooth's width (tw). D. Micro-teeth on collar chaetae, similar to those found on capillary and limbate chaetae on other portions of the body, from *S. columbiana*. Tooth length (tl) is indicated by the white bracket, and tooth width (tw) by the dashed black line.

Prior work (Merz & Woodin 2000) demonstrated that the body segments that have the most interaction with the tube wall are those with the largest diameter. The position and extent of this region of the body varied among species. Therefore, for each individual I measured the anterior–posterior length of chaetae-bearing segments associated with the broadest region of the worm (usually those segments that had diameters that were 80% or more of the largest chaetae-bearing segment) (Fig.1A).

The structures that are most likely to interact with the tube wall are the chaetae, hair-like bristles and hooks associated with most parapodia. To get an estimate of the functional size of chaetae, I measured the anterior–posterior length of a sample of the heads of hooks (chaetae with distally curved tips typically with longer shafts extending deeply into the parapodia), uncini (multi-dentate hooked chaetae, characteristic of many groups of tube-dwelling worms), or paleae (thickened or flattened chaetae) that clearly had distinct working surfaces that could be or were seen to be in contact with the tube wall or that had wear patterns that indicated regular contact with the tube wall (Merz & Woodin 2000) (Fig.1B–D). Variation in worm morphology precluded sampling a standardized number of chaetae across all samples, but in all cases I measured multiple chaetae of each type from the body segments with the broadest diameters (except when a type of chaeta occurred on only one of those segments); in most cases these measurements included 30 or more of each type of chaeta per specimen.

I also measured the length and width of toothed dentition associated with the working surface of chaetae including dentition on capillary chaetae (long tapering chaetae) in the same region on the body. I was interested in measuring the portion of the dentition free to interact with the tube surface. On multi-dentate chaetae, the teeth may be arranged in a linear anterior–posterior row, or as an interdigitated field of teeth (e.g., *Serpula columbiana,* Fig.1C,D, and numerous examples in Supporting Information, Figs. S1–S14). For those in a row, tooth length was measured as the distance from one tooth tip to the next tip in line (Fig.1C). When the surface of a chaeta was covered with teeth in offset rows (Fig.1D), tooth length was measured as the distance from the tip of one tooth to the level of the tips of adjacent teeth. In either case, tooth width was defined as the maximum width within the span covered by the defined tooth length (Fig.1C,D).

**Figure 2:**
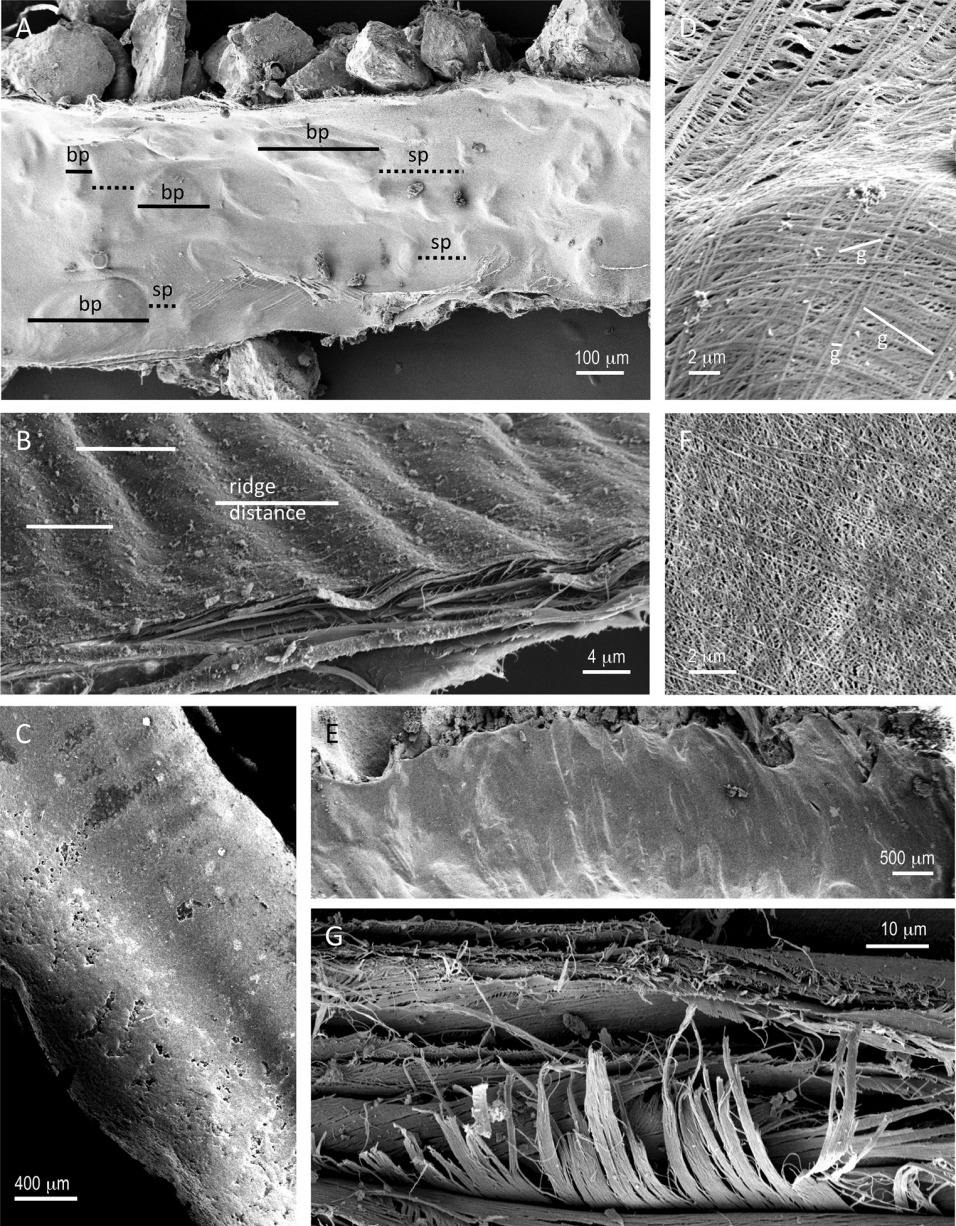
Examples of macroscopic and microscopic textures on the interior of polychaete tubes that were measured for comparison with features on the bodies of the resident worms. A. The inner surface of the tube of *Mesochaetopterus taylori*. Sedimentary particles incorporated into the outer layers of the tube produce bumps (bp, solid black lines) and spaces (sp, dashed black lines) between the bumps. B. The torn edge and internal surface of the tube of *Owenia collaris* display a series of tiny circumferential ridges. Examples of the distances measured between adjacent ridges are indicated by the solid white lines. C. The calcium carbonate tube of *Serpula columbiana*, showing variation in secretion that results in a series of internal ridges (see Fig.1C,D for a sample of this worm's chaetae and Fig.2F for a magnified view of the surface). D. Highly magnified view of the internal surface of the tube of *M. taylori* (Fig.2A) revealing the layers of strands of secreted material and the gaps between those strands. Examples of the ranges of the maximum dimension of gaps (g) formed by the innermost layer of strands are indicated by the solid white lines. E. The internal surface of the tube of the ampharetid, *Schistocomus hiltoni*. The worm has incorporated splinters of wood and other plant debris into the outer layer of the tube causing a series of internal bumps and edges. F. The inner lining of the calcium carbonate tube of the serpulid *S. columbiana* made of a fine mesh of relatively evenly sized strands. G. The torn edge of the tube of *Onuphis* sp. reveals that this structure is made of multiple layers, each with a different fiber orientation, forming a type of natural plywood that is characteristic of polychaete tubes.

After fixation and drying, tubes were opened along their long axes to reveal the macroscopic and microscopic texture of the inner lining. The lengths of bumps in the inner tube wall (which resulted from the presence of exogenous material incorporated into the outer tube layers) and the spaces between them were measured along the tube's long axis as perceived by the distortion and drape of the tube's inner lining (Fig.2A). When there were ridges in the inner tube lining (regular or semi-regular depositions on the inner layers: e.g., *Owenia collaris, S. columbiana*), I similarly measured the distance between the tops of the ridges (Fig.2B,C). Under higher magnification, I measured the diameter of strands that made up the “fabric” of the tube lining and the maximum dimension of the gaps between adjacent strands in the innermost (most recently secreted) layer of strands (Fig.2D). These measurements were made at multiple positions along a tube. Sample sizes of bumps (or ridges), spaces, strands, and gaps were typically about 30 each per specimen.

The measurements of the features on the bodies and tubes were used to define a range of sizes for that feature for a given worm. These ranges were then compared within a worm to see if there were areas of overlap that might suggest physical interaction between the worm and its tube. Lastly, comparisons among species that span the phylogeny of the group allowed me to define what constitutes the broader structural patterns and the exceptions to them.

## Results

### Tube textures at different scales

**Figure 3:**
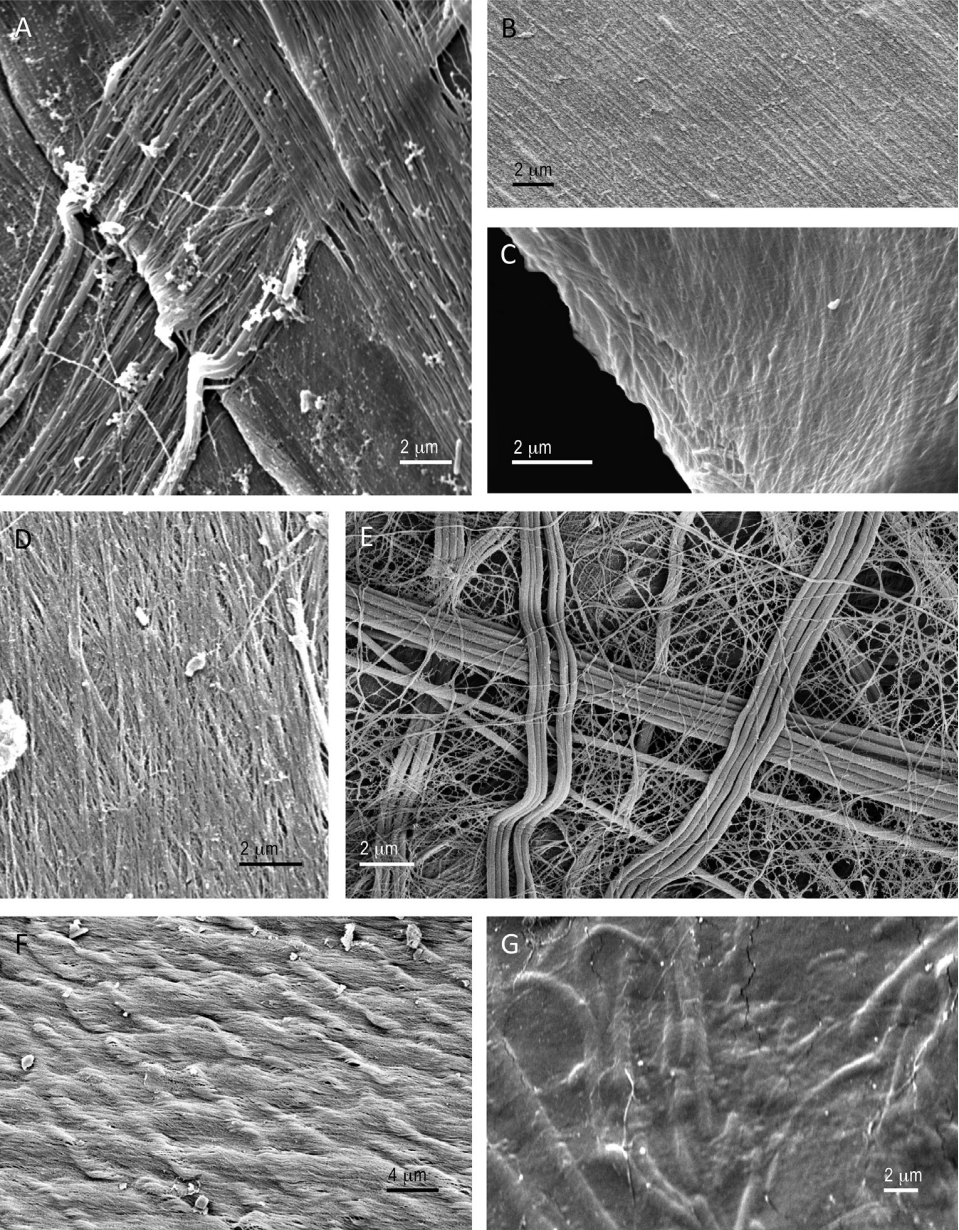
The microscopic landscape or bas-relief of the inner lining of a variety of polychaete tubes. A. The siboglinid *Oasisia* sp. deposits swaths of tube material that form a distinctive pattern of edges (see Fig. S3 for images of the associated chaetae). B. The extremely smooth inner lining of the tube of the sabellid *Eudistylia vancouveri* (see Figs.4D, S7, and S8 for images of sabellid chatae). C. The inner lining of the tube of the maldanid *Clymenella torquata* (see Fig. S12 for images of associated chaetae). D. The typical layers of densely spaced strands of the tube lining of the terebellid *Pista brevibranchiata (*see Fig. S9 for associated chaetae). E. The less organized, more open meshwork of inner tube fabric of the nereidid *Platynereis bicanaliculata* (refer to Fig. S14 for images of its chaetae). F. The unusual wrinkled or quilted inner surface of the tube of the onuphid *Diopatra ornata*. Note the exceedingly fine fibers that make up the material (see Figs.4F and S13 for chaetal surfaces). G. The alvinellid *Alvinella pompejana* lays down tube material over bacteria and thus incorporates them into its tube wall producing a fine scale rugose surface (see Fig. S2 for images of the chaetae).

For each species examined, the inner surface of the tube had a characteristic structure. On the “macro” scale (∼10 μm–1 mm), inner tube topography came from the presence of external materials that had been incorporated into the outer layer of the tube (e.g., *Mesochaetopterus taylori* and *Schistocomus hiltoni,* Figs.2A,E; also see Figs. S1–S14) or swaths of secreted material that formed a series of edges (*Oasisia* sp., Figs.3A, S3) or variations in secretion that generated nearly circumferential bands or ridges (e.g., *Owenia collaris* and *Serpula columbiana* Fig.2B,C). In most cases, the bas-relief at the micron scale (0.01–10 μm) was provided by the layers of strands of tube material (Figs.2D,F, 3A–F). These strands were typically relatively uniform in diameter and were laid down as a closely spaced irregular mesh or network (Table2; Figs.3, S1–S14). The tubes of *M. taylori* (Fig.2D) and *Platynereis bicaniculata* (Fig.3E) featured more widely spaced strands forming larger gaps. In the latter species, the strands themselves could be of very different widths.

**Table 2:** Species, evaluation of size overlap of chaetal and tube textures, and source of tube textures for 14 species whose features were confirmed by at least three specimens. Organization of families follows Weigert et al. (2014).

Family Species	Overlap at “macro” scale? (10–1000 μm+)	Source of macro texture of tube	Overlap at “micro” scale? (0.01–10 μm)	Source of micro texture of tube
Oweniidae				
*Owenia collaris*	Yes, band of micro hooks≥spaces	Sediment grains	Yes, micro hooks≤micro-ridges	Secreted micro-ridges
Chaetopteridae				
*Mesochaetopterus taylori*	Yes, chaetal heads≤spaces	Sediment grains	Yes, micro-teeth width∼=gaps	Strands form gaps
Siboglinidae				
*Oasisia* sp.	Yes, chaetal heads≤swaths	Swaths of secreted tube material	No, micro-teeth widths>gaps	Strands form gaps
Sabellaridae				
*Sabellaria cementarium*	Yes, chaetal heads≤spaces	Sediment grains	Yes, micro-teeth width≤gaps	Strands form gaps
*Idanthyrsus macropaleus*	Yes, chaetal heads≤spaces	Sediment grains	Yes, micro-teeth width≥gaps	Strands form gaps
Serpulidae				
*Serpula columbiana*	Yes, chaetal heads<ridge spaces	Secreted ridges	Yes, micro-teeth width≥gaps	Strands form gaps
Sabellidae				
*Schizobranchia insignis*	No, chaetal heads>spaces	Tiny, unknown	Yes, micro-teeth width∼=gaps	Strands form gaps
*Eudistylia vancouveri*	No, chaetal heads>spaces	Tiny, unknown	Yes, micro-teeth width∼=gaps	Strands form gaps
Terebellidae				
*Pista brevibranchiata*	Yes, chaetal heads<spaces	Sediment grains	Yes, micro-teeth width≥gaps	Strands form gaps
Pectinariidae				
*Pectinaria gouldii*	Yes, chaetal heads<spaces	Sediment grains	Yes, micro-teeth width≥gaps	Strands form gaps
Alvinellidae				
*Alvinella pompejana*	Yes, chaetal heads≤spaces	Sediment grains	Yes, micro-teeth width≥gaps and bacterial strands	Strands form gaps and bacteria incorporated in tube
Maldanidae				
*Clymenella torquata*	Yes, chaetal heads<spaces	Sediment grains	Yes, micro-teeth width≥gaps	Strands form gaps
Onuphidae				
*Diopatra ornata*	Yes, chaetal heads<spaces	Sediment grains	Yes, micro-teeth width≤wrinkles, but>gaps	Strands form gaps, wrinkles in lining
Nereididae				
*Platynereis bicanaliculata*	Yes, chaetal heads<spaces	Sediment grains	Yes, micro-teeth width∼=gaps	Strands form gaps

Three species varied from this general pattern. The inner tube of *O. collaris* had a unique surface. Unlike virtually all other species examined at similar magnification, it was difficult to perceive any “threads” of secretion, although the torn edges of tubes did show a layered fibrous nature (Fig.2B). Instead there were fine circumferential ridges that provided texture at the micron scale (Figs.2B, S1). *Diopatra ornata* produced a “quilted” or wrinkled surface from very fine threads (Figs.3E, S14) (this was also true of *Onuphis* sp., data not shown). *Alvinella pompejana* regularly incorporated much smaller exogenous items (including bacteria) that became merged into the tube lining by successive layers of tube material deposition (Figs.3G, S12).

### Worm textures at different scales

**Figure 4:**
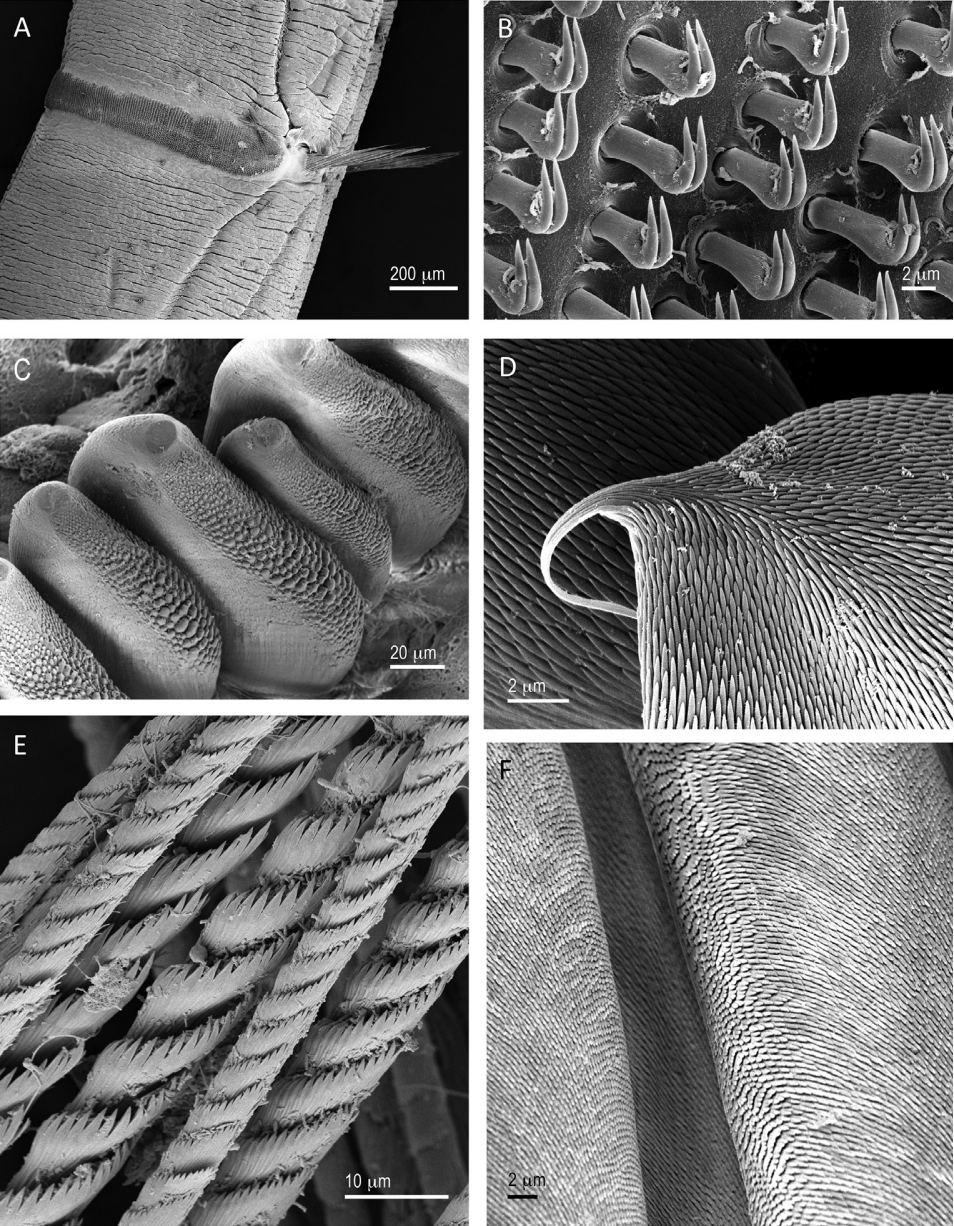
Examples of surfaces of a variety of chaetae of tube-dwelling polychaetes. A. In *Owenia collaris*, uncini occur not in a single row but in bands or tori made up of thousands of tiny individual hooks. B. Portion of a torus of chaetae and the ranks of extremely small uncini of *O. collaris* (see Fig.2B for the corresponding tube). C. Molar-like chaetae from the fourth setiger of *Mesochaetopterus taylori* with knobby, relatively blunt micro-dentition (see Figs.2A,D, and S2 for images of the tube of *M. taylori*). D. Surface of a thoracic notochaeta of the sabellid *Schizobranchia insignis* covered by thousands of micro-teeth (see Fig.3B for the inner tube lining of a closely related sabellid, *Eudistylia vancouveri*; also Figs. S7, S8). E. Abdominal capillary chaetae from the sabellarid *Idanthyrsus macropaleus* illustrating a different version of micro-dentition (see Fig. S5 for images of the tube). F. Surface of limbate chaetae of *Diopatra ornata* covered with tiny teeth (see Figs.3F and S13 for images of the interior of the tube).

In parallel with the tubes having topographic structures on different size scales, the bodies of the worms also had morphological features of different sizes that were relevant to traction between a worm and its tube. Worms’ segments and arrays of chaetae were on the scale of mm to fractions of mm, whereas the chaetal heads were usually less than 100 μm, and the sculpture and micro-dentition of chaetae were often in the range of 0.01–10 μm (Table2; Figs.4, S1–S14). Except for the hooded hooks of *P. bicanaliculata* (Fig. S14), all chaetae that were likely to interact with the tube wall had substantial micro-dentition (Figs.4, S1–S14).

### Comparison of the size overlap of textural features

For 14 species, I selected an exemplar specimen and compared the specific sizes of that worm's structures with the morphological features of its tube (Table2; Figs. S1–S14), focusing on two questions: (1) Were the chaetal heads small enough to fit within the spaces provided by the bumps (from exogenous materials) or ridges (secreted by the worm) that protruded slightly into the lumen of the tube? (2) Were the micro-teeth associated with dentate chaetae narrow enough to fit within the gaps provided by strands of secreted material or other elements of the bas-relief? From this analysis, it was clear that most of these tube-dwelling polychaetes had chaetal heads that were smaller than or equal to the spaces between bumps and secreted ridges (Table2; Figs. S1–14).

The two sabellid species, *Schizobranchia insignis* and *Eudistylia vancouveri*, were exceptions to this pattern. The older parts of their tubes consisted of many (tens to hundreds) internal layers of secreted material that had coated and smoothed out bumps originating from exogenous material in the outer layers of their tubes (Figs.3B, S7, S8). The chaetal heads of these worms were therefore substantially larger than any visible bumps or spaces (Table2; Figs. S7, S8). All chaetae associated with the widest part of these sabellids were endowed with fine dentition that overlapped the size of the fine scale bas-relief (Table2; Figs.4, S7, S8).

The tube of *Oasisia* sp. was similarly comprised of many internal layers that “erased” any bumps associated with exogenous materials. In this case the worm's secretions appeared as swaths that formed ridges that were on the size scale of its chaetal heads (Table2; Figs.3, S3). Interestingly, the micro-teeth of the uncini of this species were larger than the gaps formed by the strands within a swath of secreted material (Table2; Fig. S3).

Another variant of the macroscopic scale pattern was provided by *O. collaris*. The uncini of *O. collaris* were unusually small on an absolute scale, and uniquely occurred in bands rather than single rows (Figs.4A,B, S1). These bands of hooked chaetae were of a size that could fit into the spaces between sediment-based bumps (Table2; Fig. S1). What may be more relevant to the interaction between the body and the tube of *O. collaris* were the tiny ridges characteristic of the inner lining (Figs.2B, S1) that were approximately the same size as the individual hooks within a band of chaetae (Figs.4B, S1).

At the microscopic level, in most species there was overlap in size between the bas-relief of the tube and the fine dentition of the chaetae (Table2). I have conservatively measured micro-tooth width to evaluate this relationship, however, since all micro-teeth came to a point, it is possible that the tips of teeth could catch or snag on tube material without necessarily embedding the whole tooth (Fig.4C–F). Thus, it is likely that the micro-dentition on the chaetae of all these tube-dwelling species is capable of close physical interaction with the fine sculpture of tube surfaces.

## Discussion

### Tube lining materials

The application of secreted fibers and sheets to the interior of polychaete tubes produces a variety of linings that resemble fabrics. Close inspection of the orientation of the threads of these fabrics gives some sense of the secretion process of individual species (Figs.3, S1–S14). These patterns suggest the application of layers of tube lining as sheets (e.g., *Schizobranchia insignis,* Fig.3B) or strips (*Oasisia* sp., Fig.3A) from a secretory surface or simultaneous secretion from a series of pores as the worm turns or moves within its tube (e.g., *Mesochaetopterus taylori*,*Serpula columbiana,* Fig.2D,F); *Clymenella torquata*,*Pista brevibranchiata*,*Platynereis bicanaliculata,* Fig.3C–E; see also Figs. S1–S14). In many tubes it was possible to see the overlapping layers of nearly parallel threads whose orientation varied from one layer to the next forming a natural plywood (*sensu* Neville 1993) (e.g., *Owenia collaris,* Fig.2B, *Onuphis* sp., Fig.2G, *Oasisia* sp., Fig.3A). Natural plywoods are typical in structures that efficiently and effectively resist forces from multiple directions (e.g., insect cuticle, plant cell walls) (Wainwright et al. 1976; Neville 1993), and this construction helps explain the rigidity and robustness of polychaete tubes.

The inner tube of *O. collaris* has a unique surface of fine circumferential ridges that provide texture at the micron scale (Figs.2B, S1) that closely match the size of that species’ equally unique micro-hooks (Fig.4A,B). In addition to the texture that the ridges provide, they may also contribute to the flexibility of this tube. *Owenia collaris* lives with its tube partially extending into the water column above the water-sediment interface, and the worm feeds either by exposing its mucus-covered feeding crown to the surrounding water, or by bending its tube over and sweeping the surface of the surrounding sediment with its tentacles (Dales 1957). The micro-ridges may allow local extension and contraction of the tube as it is bent by the worm's activities.

The texture of the inner lining of the tube formed by *Diopatra ornata* has a quilted appearance made up of exceedingly fine threads that largely run in parallel, but have the appearance of being scrunched or gathered together to form a wrinkled surface (Figs.3, S13). Whether this pattern is a result of the way the material is secreted or is the result of patterning after it is in position but before hardening in seawater is not possible to tell at this point. Given that this unusual surface was also evident in *Onuphis* sp. (data not shown), it suggests that this lineage of Errantia (Struck et al. 2011; Weigert et al. 2014) may have a divergent mode of generating tubes.

The tubes of *P. bicanaliculata* provide a dramatic contrast to the orderly, densely packed fiber arrays of tubes of virtually all other species examined (Fig.3E). Individual threads are in at least two different size classes, and their distribution suggests applications of the secreted material as single or a few threads rather than as sheets or simultaneous arrays of threads. This accords well with Daly's (1973) description of the construction of the tube by *P. dumerilii*. Tube dwelling is a relatively unusual life style within the Phyllodocida, a crown group recognized by both morphological and molecular evidence (Rouse & Fauchald 1997; Struck et al. 2007; Weigert et al. 2014). The unusual tube construction of *P. bicanaliculata* could be taken as evidence of the independent origin of tube dwelling in this lineage.

### Are worms like geckos?

The fine dentition of the surfaces of chaetae of tube-dwelling polychaetes is reminiscent of the fine setae associated with gecko, insect, and spider feet (Artz et al. 2003) that are credited with allowing those groups to walk up vertical walls or hang up-side down from smooth surfaces by virtue of a combination of van der Waals forces and capillary action (Autumn et al. 2002; Artz et al. 2003; Kwak & Kim 2010). The present morphological study was not designed to fully examine the question of whether the dentition of tube-dwelling polychaete chaetae acts in the same way as these terrestrially based systems; however, it is possible to recognize some specific similarities and differences. They are alike in that polychaete chaetae are on appendages that are used for climbing up what can be nearly vertical surfaces (the insides of tubes, Table1). Chaetal dentition is also part of a complex hierarchical morphology that ultimately also provides a finely divided surface that approaches the critical size suggested to be necessary by theory (Autumn et al. 2002) and exhibited by the convergence of the setae of reptiles, insects, and chelicerates (0.2–5.0 μm, Artz et al. 2003). Van der Waals forces that are responsible (at least in part) for gecko adhesion could be applicable to systems in either air or water depending on the hydrophilic/hydrophobic character of the materials and characteristics of the surface topography (Autumn 2006; Ditsche et al. 2014).

The performance capabilities of the animals are different—for example, geckos or insects can walk up vertical sheets of clean glass, but tube-dwelling polychaetes transferred to clean glass or plastic tubes that have the same diameter as their original tubes cannot easily maintain a vertical position in the tube (in water). If worms are allowed to reside in artificial tubes and lay down inner tube linings then they may maintain their position well (Woodin et al. 2003). The isolated toes and setae of geckos adhere strongly to glass surfaces, indicating that their adhesion is effective even without the active participation of the animal (Autumn et al. 2002). In contrast, anesthetized worms in their own tubes or in artificial tubes do not maintain their position, indicating that to do so requires active participation by the worms (Woodin & Merz 1987; Merz & Woodin 2000). In morphological terms, the micro-teeth of polychaete chaetae are typically pointed, whereas the setae of geckos, spiders, and insects have fine spatulate tips (Artz 2003) that are considered to be critical in providing the flexible surface needed for maximum contact with the surface necessary to achieve adhesion by van der Waals forces and capillary action (Rizzo et al. 2006). In addition, the spatulate tip of a gecko or insect seta is attached to the shaft of the seta by an even narrower neck, so that the spatulate tip itself can adjust its angle to local variations in substrate topography (Rizzo et al. 2006). There is no morphological indication that the dentition of polychaete chaetae is similarly flexible. Given the differences in ability and architecture between geckos and insects compared to tube-dwelling polychaetes it seems unlikely that the latter are using van der Waals forces or capillary action to gain traction with their tube walls.

If worms do not move within their tubes like geckos, how do they successfully grip the walls of their tubes and how do they quickly release that attachment when rapid retraction is necessary? The hydrostatic skeleton of polychaetes allows regional changes in body diameter. Maximizing worm diameter presses parapodia and their associated chaetae into the tube wall, engaging the chaetae and maximizing friction. Constricting body diameter pulls the chaetae away from the tube wall, diminishing traction and allowing rapid withdrawal. In addition, within parapodia there are intrinsic muscles that can retract or extend chaetae or chaetal bundles and adjust their position on a much finer scale (Tzetlin & Filippova 2005).

### Chaetal sculpturing at two size scales

All tube-dwelling polychaetes have uncini or hooked chaetae; in all species in which the function of these chaetae has been studied, they have been demonstrated to play a role in anchoring the worms in their tubes. What has been less appreciated is the role of micro-dentition on hooks, but also on less well-studied varieties of chaetae (e.g., capillary chaetae, palae, limbate chaetae, etc.). The general pattern in tube-dwelling polychaetes is that there are two scales at which there is a size match between morphological features of the bodies of the worms and the tubes they inhabit. At a macroscopic or nearly macroscopic scale (∼10 μm–1 mm), the chaetal heads that interact with the tube wall are small enough to fit within the spaces between inward projections of exogenous materials incorporated into the tube wall (bumps) or between ridges of secretions generated on the interior surface. At a finer scale (0.01–10 μm) there is a second overlap in size, usually between the dentition on the surfaces of chaetae that interact with the tube walls and the texture provided by the secreted strands and gaps of the inner lining and from microscopic inclusions. This textural overlap at two size ranges may be analogous to the way in which a ladder constructed by humans also has two size matches—one between the spacing of the rungs and the length of human leg bones, and a second between the anti-slip surfaces of the rungs and the tread of shoe soles. Each is necessary for us to climb up and down efficiently with relatively sure footing.

## Supporting Information

Additional Supporting information may be found in the online version of this article. Comparisons of the macro- and microscopic features of the bodies and tube linings for fourteen species of polychaetes are illustrated in scanning electron micrographs in Figs. S1–S14. Each figure depicts the body and tube of a single species at increasing levels of magnification. Within each species the illustrations of features may have been drawn from more than one individual in order to provide the best view of the structure. To aid in recognizing patterns of overlap in the size of features between the bodies and tubes, a visual summary of the ranges of sizes for a variety of morphological features on the body and tube of a single representative of each species is provided. Obviously, different sized individuals within a species would have features of different sizes, therefore, these diagrams are useful in seeing where the size of features overlap within a particular individual or for broad comparisons of the general patterns of overlap amongst species, but not for comparing specific size ranges.

Fig S1 *Owenia collaris* (Oweniidae): body and tube. **A**. Anterior segment showing notopodial capillary chaetae and neuropodial torus of tiny hooks. **B**. Tips of capillary chaetae with micro-teeth. **C**. Section of a field of hooks within a torus. **D**. Longitudinal section of tube with circumferential ridges and larger bumps associated with sediment grains incorporated into the exterior portion of the tube. **E**. Inner tube surface of tiny ridges. The size ranges for a single worm (0.9 mm diam.) indicate that the anterior–posterior span of the chaetal rows (ch row) overlap the sizes of the sediment-based bumps (bp) and the spaces (sp) between bumps. Tooth widths (tw) of the hooks are much smaller than other measured features, but tooth lengths (tl) overlap in size with the tube's ridges (ridges).

Fig S2 *Mesochaetopterus taylori* (Chaetopteridae): body and tube. **A**. Anterior segments of worm. **B**. Uncini from tenth setiger. **C**. Lanceolate chaeta from anterior segment. **D**. Surface of lanceolate chaeta. **E**. Knob-like chaeta from fourth setiger. **F**. Surface of knob-like chaeta. **G**. Longitudinal section of tube showing internal texture provided by encrusted external sediment. **H**. Inner tube lining. **I**. Texture of inner tube lining. The size ranges for a single worm (1.3 mm diam.) indicate that the size of chaetal heads (ch) of uncini and knob-like chaetae overlaps the size of spaces (sp) between bumps (bp) caused by sediment incorporated into the external tube wall. Segments (seg) are larger than these bumps. Chaetal dentition as represented by tooth widths (tw) and lengths (tl) overlaps the gaps (g) formed by the strands (st) of the tube lining.

Fig S3 *Oasisia* sp. (Siboglinidae): body and tube. **A**. Anterior region and collar of worm. **B**. Opisthosoma with rows of uncini. **C**. Individual multidentate uncini from opisthosoma. **D**. Longitudinal section of tube. **E**. Inner tube lining. **F**. Swaths of secreted tube lining that provide the microstructure of inner tube. The size ranges for a single worm (2.1 mm diam.) indicate that the size of chaetal heads (ch) are much smaller than the length of segments (seg) and overlap the size of swaths (swth) of secreted tube material associated with the inner tube texture. The tooth widths (tw) and lengths (tl) of chaetae are larger than the size of gaps (g) formed by the strands (st) of the tube lining.

Fig S4 *Sabellaria cementarium* (Sabellaridae): body and tube. **A**. Anterior, parathoracic and abdominal segments. **B**. Parathoracic paleae. **C**. Row of abdominal uncini. **D**. Dentition on palea. **E**. Dentition on uncini. **F**. Longitudinal section of tube with internal texture provided by incorporated sediment. **G**. Microstructure of tube lining. The size ranges for a single worm (1.0 mm diam.) show that chaetal heads (ch) of uncini and paleae are smaller than or equal to the spaces (sp) between the bumps (bp) caused by sediment. The grains are usually smaller than segment lengths (seg). Chaetal dentition has a broad range of tooth lengths (tl) but a narrower range of widths (tw) that overlap the size of gaps (g) formed by the strands (st) of the tube lining. Capillary chaetae (data not shown) resemble those of *Idanthyrsus macropaleus*, Fig. S5.

Fig S5 *Idanthyrsus macropaleus* (Sabellaridae): body and tube. **A**. Whole worm. **B**. Abdominal capillary chaetae. **C**. Lateral view of abdominal uncini. **D**. Micro-teeth on palea (similar to those found in *Sabellaria cementarium*, Fig. S4). **E**. Inner tube surface. **F**. Microstructure of tube lining. The size ranges for a single worm (1.4 mm diam.) indicate that chaetal heads (ch) of uncini and paleae fall in the middle of the size range of spaces (sp) existing between the bumps (bp) caused by sediment particles. The size range of bumps is exceptionally large, overlapping the size of chaetal dentition, chaetal heads (ch), and segments (seg). Chaetal dentition has broad size ranges of tooth lengths (tl) and widths (tw) that barely (former) and broadly (latter) overlap the size of gaps (g) formed by the strands (st) of the tube lining.

Fig S6 *Serpula columbiana* (Serpulidae): body and tube. **A**. Thoracic collar chaetae. **B**. Rows of abdominal uncini. **C**. Thoracic uncini. **D**. Micro-teeth on collar chaetae, similar to those found on capillary and limbate chaetae on other portions of the body. **E**. Inner tube surface. **F**. Microstructure of tube lining. The size ranges for a single worm (1.8 mm diam.) indicate that chaetal heads (ch) of uncini are smaller than the spaces (rsp) between the ridges (r) deposited by the worm. The length of the ridges and the distance between them is smaller than the length of anterior segments (seg). The smaller portion of the size range of tooth lengths (tl) overlaps that of the largest tooth widths (tw). The smaller tooth widths overlap in size with the gaps (g) formed by the strands (st) of the tube lining.

Fig S7 *Schizobranchia insignis* (Sabellidae): body and tube. **A**. Whole worm *sans* feeding crown. **B**. Thoracic notopodium. **C**. Thoracic notopodial chaetae. **D**. Thoracic uncinus. **E**. Micro-teeth typical of chaetal surfaces of the thorax. **F**. Longitudinal section and cut edges of tube. **G**. Tiny bumps typical of anterior portion of tube lining. **H**. Microstructure of tube lining. Size ranges for a single worm (0.9 mm diam.) indicate that segments (seg) and chaetal heads (ch) of uncini, companion chaetae (see Fig. S8), and thoracic notopodial chaetae are larger than the size of spaces (sp) and bumps (bp) associated with the inner tube. The surfaces of all chaetae examined were adorned with micro-teeth with tooth lengths (tl) and widths (tw) that overlapped the size of gaps (g) formed by the strands (st) of the tube lining. Arrangement and morphology is very similar to *Eudistylia vancouveri*, Fig. S8.

Fig S8 *Eudistylia vancouveri* (Sabellidae): body and tube. **A**. Thorax. **B**. Thoracic uncini and companion chaetae. **C**. Thoracic uncini. **D**. Companion chaetae. **E**. Tube section with smooth inner layer in contrast with rough outer surface. **F**. Small bumps typical of anterior inner tube lining. **G**. Microstructure of tube lining. The size ranges for a single worm (4.4 mm diam.) show that the lengths of the worm's segments (seg) and the chaetal heads (ch) of thoracic chaetae are larger than the tiny spaces (sp) and bumps (bp) of the inner texture. The surfaces of all chaetae were adorned with micro-teeth that had tooth widths (tw) and lengths (tl) that overlapped the size of gaps (g) formed by the strands (st) of the tube lining. Other thoracic chaetae were like those of *Schizobranchia insignis* (Fig. S7B–E).

Fig S9 *Pista brevibranchiata* (Terebellidae): body and tube. **A**. Anterior segments. **B**. Anterior notopodium. **C**. Anterior uncini. **D**. Micro-teeth on anterior notopodial chaetae. **E**. Longitudinal section of tube showing internal texture provided by sediment grains incorporated into the exterior portion of the tube. **F**. Microstructure of tube lining with an interruption of the normally even surface. The size ranges for a single worm (2.6 mm diam.) indicate that chaetal heads (ch) of uncini are smaller than the spaces (sp) or bumps (bp) caused by sediment grains that are themselves smaller than the length of the worm's segments (seg). Chaetal dentition has a broad range of tooth lengths (tl) and widths (tw). The smaller dentition overlaps the size of gaps (g) formed by the strands (st) of the tube lining.

Fig S10 *Pectinaria gouldii* (Pectinariidae): body and tube. **A**. Mid-body parapodium. **B**. Texture of capillary chaeta. **C**. Mid-body row of uncini. **D**. Uncini. **E**. Fractured top edge of tube. **F**. Fractured edge of foamy glue that attaches sand grains. **G**. Texture of inner tube lining including holes in the fabric perhaps caused by the worm's uncini (compare size and pattern of the dentition of the uncini and the holes). The size ranges for a single worm (5.0 mm diam.) indicate that chaetal heads (ch) of uncini are smaller than the spaces (sp) or bumps (bp) caused by sand grains which are themselves smaller that the length of the worm's segments (seg). Chaetal dentition has a broad range of tooth lengths (tl) and widths (tw). But only the smaller range of tooth widths overlaps the size of gaps (g) formed by the strands (st) of the tube lining.

Fig S11 *Alvinella pompejana* (Alvinellidae): body and tube. **A**. Anterior chaetal spines. **B**. Worn surface of spines. **C**. Mid-body neuropodium with uncini. **D**. Mid-body uncini. **E**. Mid-body notopodium with capillary chaetae. **F**. Serrated capillary chaetae. **G**. Inner tube lining. **H**. Bacteria incorporated into tube lining. The size ranges for a single worm (4.0 mm diam.) indicate that segment size (seg) falls in the middle of the range of bumps (bp) and spaces (sp) produced by incorporated material; chaetal heads (ch) overlap the smaller portion of this range. Tooth widths (tw) and lengths (tl) cover a large size range and overlap with the gaps (g) formed by the strands (st) of the tube lining. The rugosity offered by the bacteria (bact) incorporated into the tube wall is on a size scale similar to the finest dentition.

Fig S12 *Clymenella* torquata (Maldanidae): body and tube. **A**. Posterior parapodium with capillary chaetae and row of uncini. **B**. Row of uncini. **C**. Shafts of capillary chaetae. **D**. Uncinus. **E**. Surface of capillary chaeta. **F**. Longitudinal section of tube. **G**. Tilted view of tube lining to show texture. **H**. Surface of lining. The size ranges of a single worm (1.8 mm diam.) show that the chaetal heads (ch) of uncini are smaller than the bumps (bp) or spaces (sp) caused by sediment incorporated into the tube's exterior. The worm's segments (seg) are much larger than these sand grains. Chaetal dentition, as represented by tooth widths (tw) and lengths (tl) of uncini and capillary chaetae, has a broad size range. The gaps (g) formed by the strands (st) of the tube lining overlap in size with the smaller portion of the range of chaetal dentition.

Fig S13 *Diopatra ornata* (Onuphidae): body and tube. **A**. Mid-body neuropodium. **B**. Hooded hook. **C**. Pectinate chaeta. **D**. Surface dentition of limbate chaeta. (B,C, and D are from mid-body neuropodia). **E**. Longitudinal section of tube. **F**. Inner tube lining. **G**. Wrinkled texture of inner lining. The size ranges of a single worm (4.2 mm diam.) show that the chaetal heads (ch) of hooks and pectinate chaetae are smaller than the bumps (bp) and spaces (sp) associated with materials incorporated into the tube's exterior. The worm's segments are generally larger than these features but slightly overlap the size of spaces. Tooth widths (tw) and lengths (tl) associated with the various chaetae overlap the size range of the wrinkles (wr) that form the dominant texture of the inner tube lining. The gaps (g) formed by strands (st) are smaller than the chaetal dentition.

Fig S14 *Platynereis bicanaliculata* (Nereididae): body and tube. **A**. Anterior segments of worm. **B**. Hooks. **C**. Compound (jointed) falcate spinagers. **D**. Compound (jointed) falcigers. **E**. Longitudinal section of tube. **F**. Inner surface of tube lining. **G**. Texture of inner tube. The size ranges of a single worm (1.1 mm diam.) show that the chaetal heads (ch) of hooks and falcigers are smaller than the spaces (sp) and bumps (bp) formed by the inclusion of algae, organic and inorganic materials in the outer layers of the tube. The size of these materials is similar to the length of the worm's segments (seg). Strands (st) of secreted material form gaps (g) that have a large range of sizes, overlapping the size of chaetal heads and chaetal dentition characterized by tooth lengths (tl) and tooth widths (tw).
