# Supplementary material for: Textures and traction: how tube-dwelling polychaetes get a leg up
Source: Invertebr Biol. 2015 Mar 3;134(1):61–77. doi: 10.1111/ivb.12079 (PMC4375521; doi:10.1111/ivb.12079)
Supplement: Fig S1 — Owenia collaris (Oweniidae): body and tube. A. Anterior segment showing notopodial capillary chaetae and neuropodial torus of tiny hooks. B. Tips of capillary chaetae with micro-teeth. C. Section of a field of hooks within a torus. D. Longitudinal section of tube with circumferential ridges and larger bumps associated with sediment grains incorporated into the exterior portion of the tube. E. Inner tube surface of tiny ridges. The size ranges for a single worm (0.9 mm diam.) indicate that the anterior–posterior span of the chaetal rows (ch row) overlap the sizes of the sediment-based bumps (bp) and the spaces (sp) between bumps. Tooth widths (tw) of the hooks are much smaller than other measured features, but tooth lengths (tl) overlap in size with the tube's ridges (ridges). [file ivb0134-0061-sd1.pdf]

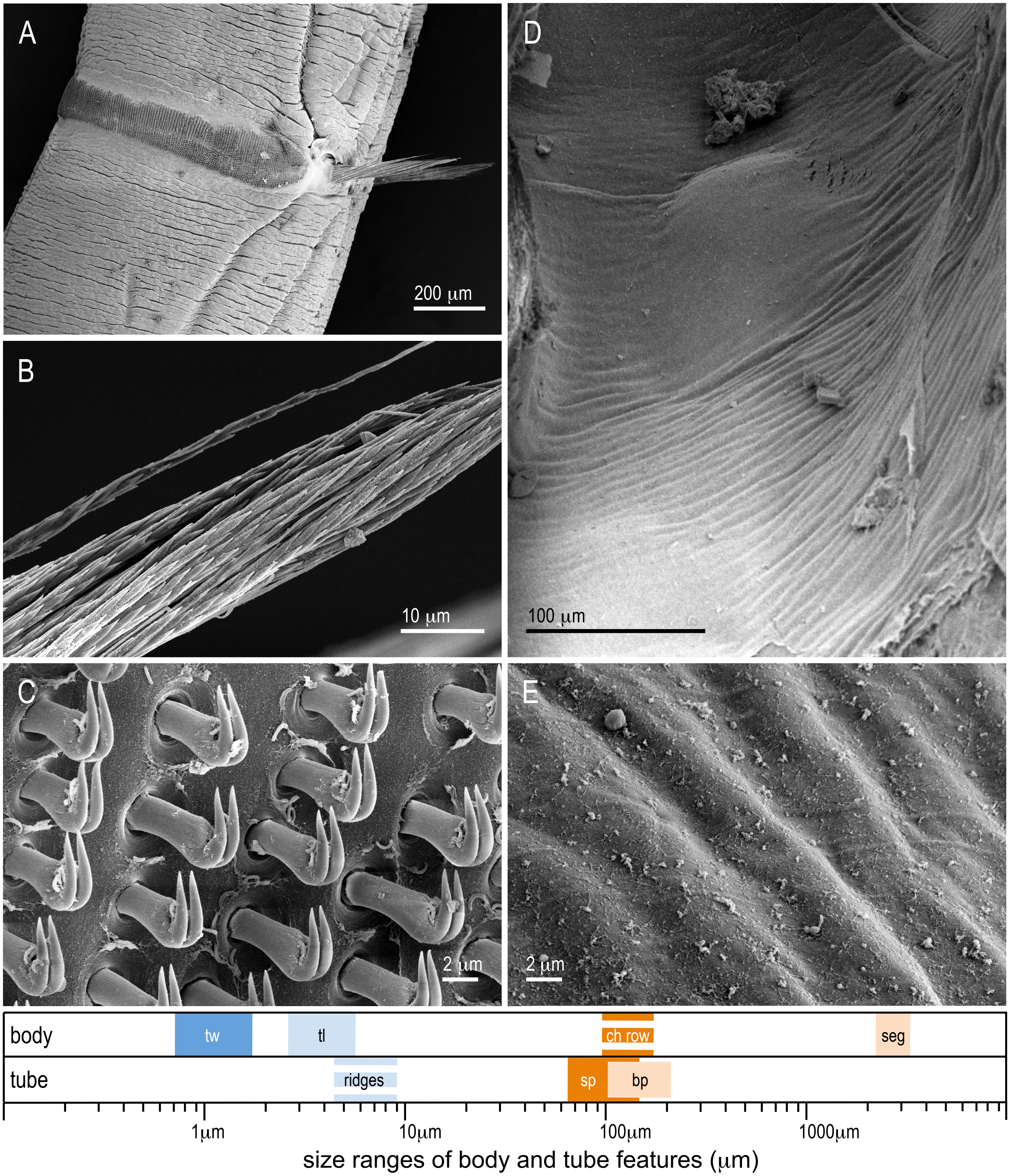

Fig. S1. *Owenia collaris* (Oweniidae): body and tube. **A.** Anterior segment showing notopodial capillary chaetae and neuropodial torus of tiny hooks. **B.** Tips of capillary chaetae with microteeth. **C.** Section of a field of hooks within a torus. **D.** Longitudinal section of tube with circumferential ridges and larger bumps associated with sediment grains incorporated into the exterior portion of the tube. **E.** Inner tube surface of tiny ridges. The size ranges for a single worm (0.9 mm diam.) indicate that the anterior-posterior span of the chaetal rows (ch row) overlap the sizes of the sediment-based bumps (bp) and the spaces (sp) between bumps. Tooth widths (tw) of the hooks are much smaller than other measured features, but tooth lengths (tl) overlap in size with the tube's ridges (ridges).
