# Supplementary material for: Textures and traction: how tube-dwelling polychaetes get a leg up
Source: Invertebr Biol. 2015 Mar 3;134(1):61–77. doi: 10.1111/ivb.12079 (PMC4375521; doi:10.1111/ivb.12079)
Supplement: Fig S2 — Mesochaetopterus taylori (Chaetopteridae): body and tube. A. Anterior segments of worm. B. Uncini from tenth setiger. C. Lanceolate chaeta from anterior segment. D. Surface of lanceolate chaeta. E. Knob-like chaeta from fourth setiger. F. Surface of knob-like chaeta. G. Longitudinal section of tube showing internal texture provided by encrusted external sediment. H. Inner tube lining. I. Texture of inner tube lining. The size ranges for a single worm (1.3 mm diam.) indicate that the size of chaetal heads (ch) of uncini and knob-like chaetae overlaps the size of spaces (sp) between bumps (bp) caused by sediment incorporated into the external tube wall. Segments (seg) are larger than these bumps. Chaetal dentition as represented by tooth widths (tw) and lengths (tl) overlaps the gaps (g) formed by the strands (st) of the tube lining. [file ivb0134-0061-sd2.pdf]

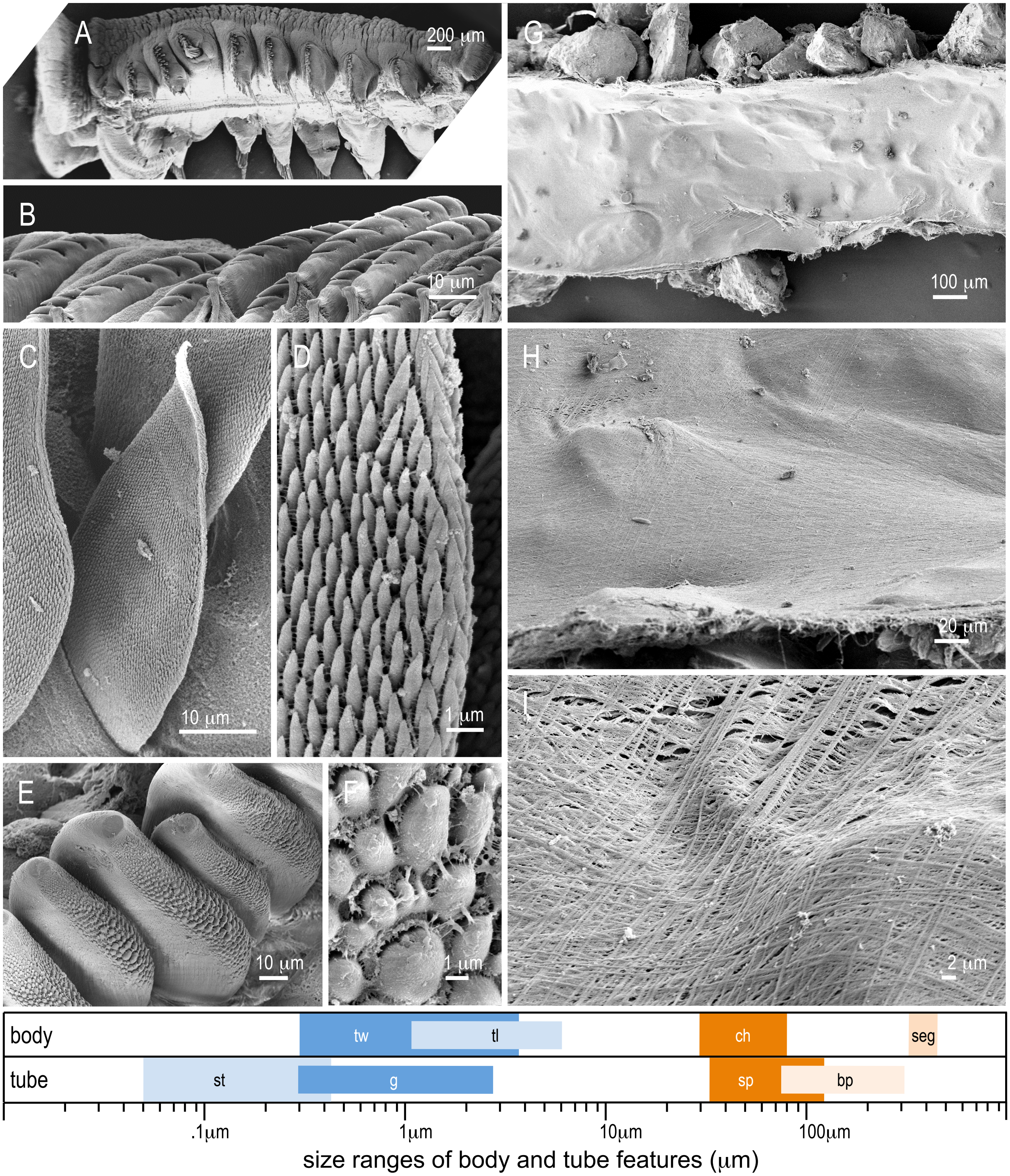

Fig. S2. *Mesochaetopterus taylori* (Chaetopteridae): body and tube. **A**. Anterior segments of worm. **B**. Uncini from tenth setiger. **C**. Lanceolate chaeta from anterior segment. **D**. Surface of lanceolate chaeta. **E**. Knob-like chaeta from fourth setiger. **F**. Surface of knob-like chaeta. **G**. Longitudinal section of tube showing internal texture provided by encrusted external sediment. **H**. Inner tube lining. **I**. Texture of inner tube lining. The size ranges for a single worm (1.3 mm diam.) indicate that the size of chaetal heads (ch) of uncini and knob-like chaetae overlaps the size of spaces (sp) between bumps (bp) caused by sediment incorporated into the external tube wall. Segments (seg) are larger than these bumps. Chaetal dentition as represented by tooth widths (tw) and lengths (tl) overlaps the gaps (g) formed by the strands (st) of the tube lining.
