# Supplementary material for: Textures and traction: how tube-dwelling polychaetes get a leg up
Source: Invertebr Biol. 2015 Mar 3;134(1):61–77. doi: 10.1111/ivb.12079 (PMC4375521; doi:10.1111/ivb.12079)
Supplement: Fig S3 — Oasisia sp. (Siboglinidae): body and tube. A. Anterior region and collar of worm. B. Opisthosoma with rows of uncini. C. Individual multidentate uncini from opisthosoma. D. Longitudinal section of tube. E. Inner tube lining. F. Swaths of secreted tube lining that provide the microstructure of inner tube. The size ranges for a single worm (2.1 mm diam.) indicate that the size of chaetal heads (ch) are much smaller than the length of segments (seg) and overlap the size of swaths (swth) of secreted tube material associated with the inner tube texture. The tooth widths (tw) and lengths (tl) of chaetae are larger than the size of gaps (g) formed by the strands (st) of the tube lining. [file ivb0134-0061-sd3.pdf]

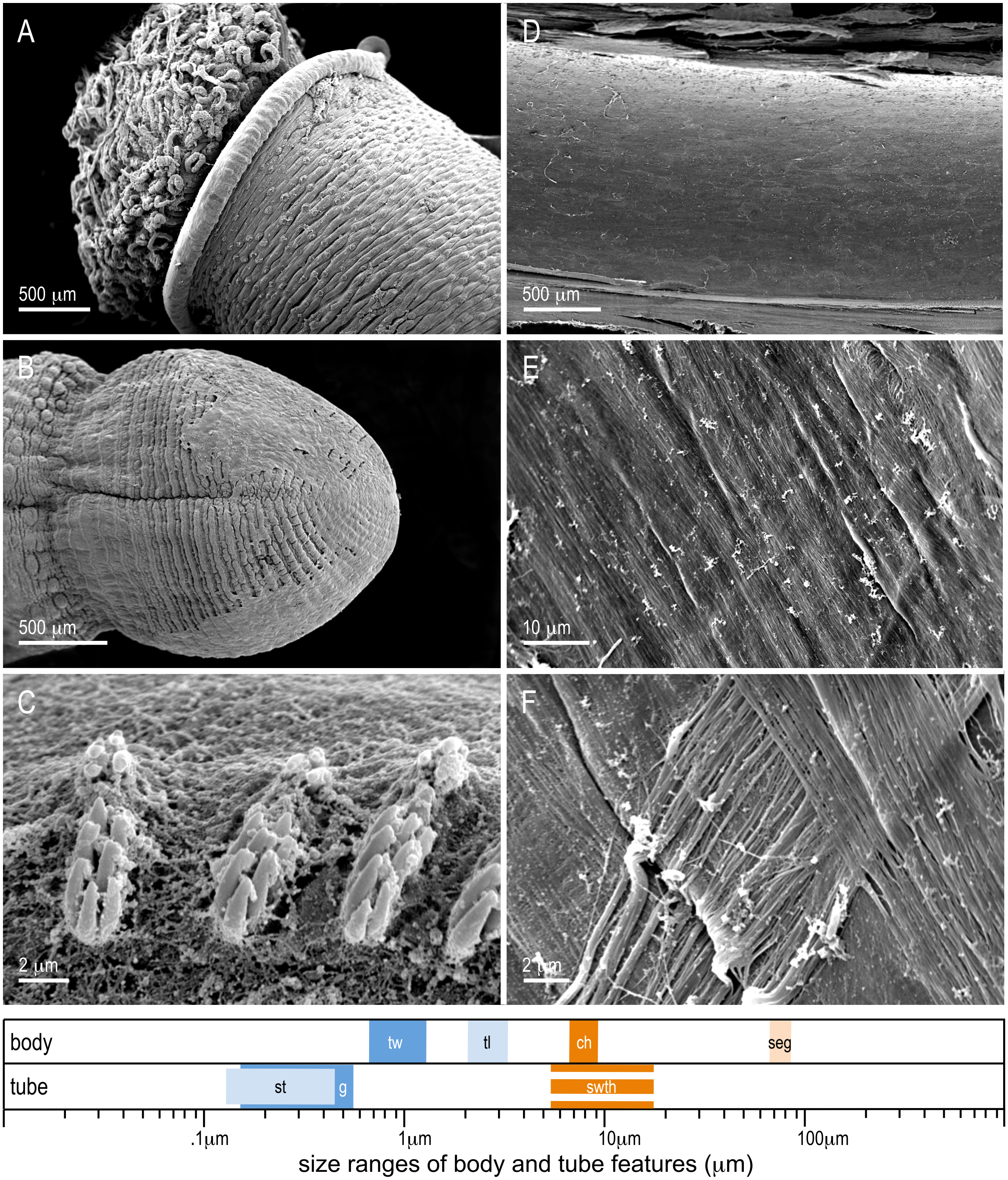

Fig. S3. *Oasisia* sp. (Siboglinidae): body and tube. **A.** Anterior region and collar of worm. **B.** Opisthosoma with rows of uncini. **C.** Individual multidentate uncini from opisthosoma. **D.** Longitudinal section of tube. **E.** Inner tube lining. **F.** Swaths of secreted tube lining that provide the microstructure of inner tube. The size ranges for a single worm (2.1 mm diam.) indicate that the size of chaetal heads (ch) are much smaller than the length of segments (seg) and overlap the size of swaths (swth) of secreted tube material associated with the inner tube texture. The tooth widths (tw) and lengths (tl) of chaetae are larger than the size of gaps (g) formed by the strands (st) of the tube lining.
