# Supplementary material for: Textures and traction: how tube-dwelling polychaetes get a leg up
Source: Invertebr Biol. 2015 Mar 3;134(1):61–77. doi: 10.1111/ivb.12079 (PMC4375521; doi:10.1111/ivb.12079)
Supplement: Fig S4 — Sabellaria cementarium (Sabellaridae): body and tube. A. Anterior, parathoracic and abdominal segments. B. Parathoracic paleae. C. Row of abdominal uncini. D. Dentition on palea. E. Dentition on uncini. F. Longitudinal section of tube with internal texture provided by incorporated sediment. G. Microstructure of tube lining. The size ranges for a single worm (1.0 mm diam.) show that chaetal heads (ch) of uncini and paleae are smaller than or equal to the spaces (sp) between the bumps (bp) caused by sediment. The grains are usually smaller than segment lengths (seg). Chaetal dentition has a broad range of tooth lengths (tl) but a narrower range of widths (tw) that overlap the size of gaps (g) formed by the strands (st) of the tube lining. Capillary chaetae (data not shown) resemble those of Idanthyrsus macropaleus, Fig. S5. [file ivb0134-0061-sd4.pdf]

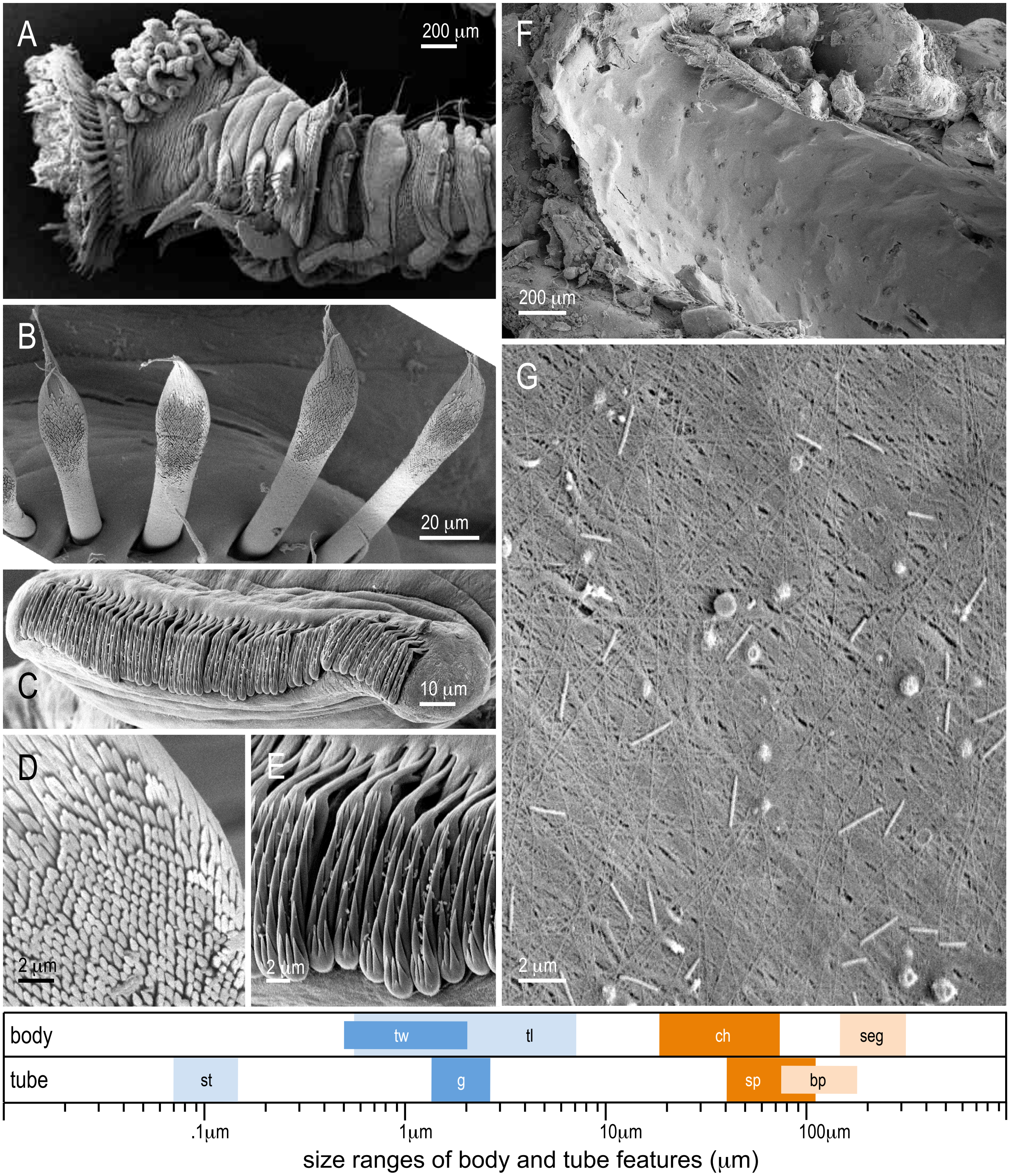

Fig. S4. *Sabellaria cementarium* (Sabellaridae): body and tube. **A.** Anterior, parathoracic and abdominal segments. **B.** Parathoracic palea. **C.** Row of abdominal uncini. **D.** Dentition on palea. **E.** Dentition on uncini. **F.** Longitudinal section of tube with internal texture provided by incorporated sediment. **G.** Microstructure of tube lining. The size ranges for a single worm (1.0 mm diam.) show that chaetal heads (ch) of uncini and palea are smaller than or equal to the spaces (sp) between the bumps (bp) caused by sediment. The grains are usually smaller than segment lengths (seg). Chaetal dentition has a broad range of tooth lengths (tl) but a narrower range of widths (tw) that overlap the size of gaps (g) formed by the strands (st) of the tube lining. Capillary chaetae (not shown) resemble those of *Idanthyrsus macropalea*, Fig. S5.
