# Supplementary material for: Textures and traction: how tube-dwelling polychaetes get a leg up
Source: Invertebr Biol. 2015 Mar 3;134(1):61–77. doi: 10.1111/ivb.12079 (PMC4375521; doi:10.1111/ivb.12079)
Supplement: Fig S5 — Idanthyrsus macropaleus (Sabellaridae): body and tube. A. Whole worm. B. Abdominal capillary chaetae. C. Lateral view of abdominal uncini. D. Micro-teeth on palea (similar to those found in Sabellaria cementarium, Fig. S4). E. Inner tube surface. F. Microstructure of tube lining. The size ranges for a single worm (1.4 mm diam.) indicate that chaetal heads (ch) of uncini and paleae fall in the middle of the size range of spaces (sp) existing between the bumps (bp) caused by sediment particles. The size range of bumps is exceptionally large, overlapping the size of chaetal dentition, chaetal heads (ch), and segments (seg). Chaetal dentition has broad size ranges of tooth lengths (tl) and widths (tw) that barely (former) and broadly (latter) overlap the size of gaps (g) formed by the strands (st) of the tube lining. [file ivb0134-0061-sd5.pdf]

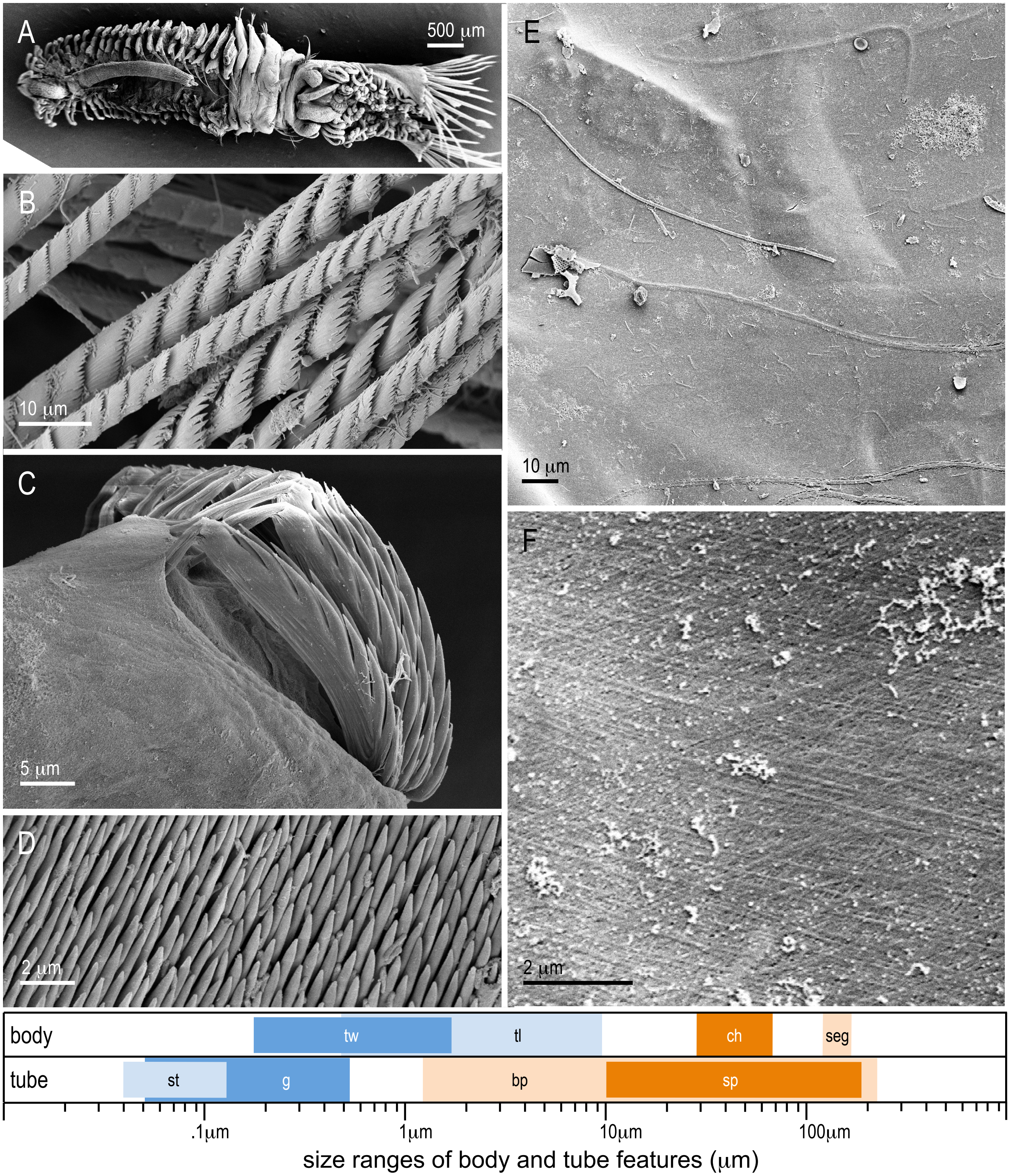

Fig. S5. *Idanthyrsus macropaleus* (Sabellaridae): body and tube. **A.** Whole worm. **B.** Abdominal capillary chaetae. **C.** Lateral view of abdominal uncini. **D.** Microteeth on palea (similar to those found in *S. cementarium*, Fig. S4). **E.** Inner tube surface. **F.** Microstructure of tube lining. The size ranges for a single worm (1.4 mm diam.) indicate that chaetal heads (ch) of uncini and paleae fall in the middle of the size range of spaces (sp) existing between the bumps (bp) caused by sediment particles. The size range of bumps is exceptionally large, overlapping the size of chaetal dentition, chaetal heads (ch) and segments (seg). Chaetal dentition has broad size ranges of tooth lengths (tl) and widths (tw) that barely (former) and broadly (latter) overlap the size of gaps (g) formed by the strands (st) of the tube lining.
