# Supplementary material for: Textures and traction: how tube-dwelling polychaetes get a leg up
Source: Invertebr Biol. 2015 Mar 3;134(1):61–77. doi: 10.1111/ivb.12079 (PMC4375521; doi:10.1111/ivb.12079)
Supplement: Fig S6 — Serpula columbiana (Serpulidae): body and tube. A. Thoracic collar chaetae. B. Rows of abdominal uncini. C. Thoracic uncini. D. Micro-teeth on collar chaetae, similar to those found on capillary and limbate chaetae on other portions of the body. E. Inner tube surface. F. Microstructure of tube lining. The size ranges for a single worm (1.8 mm diam.) indicate that chaetal heads (ch) of uncini are smaller than the spaces (rsp) between the ridges (r) deposited by the worm. The length of the ridges and the distance between them is smaller than the length of anterior segments (seg). The smaller portion of the size range of tooth lengths (tl) overlaps that of the largest tooth widths (tw). The smaller tooth widths overlap in size with the gaps (g) formed by the strands (st) of the tube lining. [file ivb0134-0061-sd6.pdf]

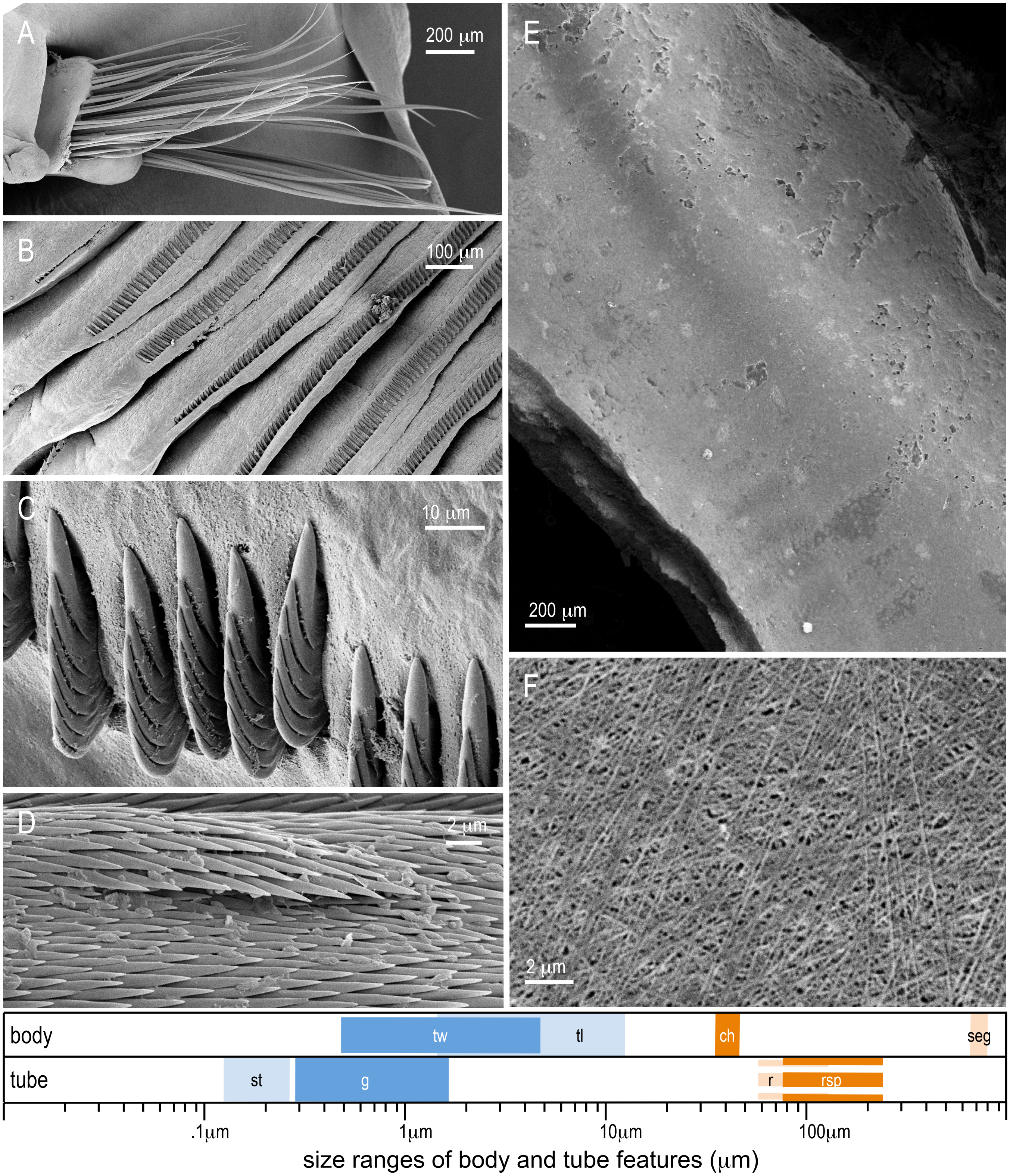

Fig. S6. *Serpula columbiana* (Serpulidae): body and tube. **A.** Thoracic collar chaetae. **B.** Rows of abdominal uncini. **C.** Thoracic uncini. **D.** Microteeth on collar chaetae, similar to those found on capillary and limbate chaetae on other portions of the body. **E.** Inner tube surface. **F.** Microstructure of tube lining. The size ranges for a single worm (1.8 mm diam.) indicate that chaetal heads (ch) of uncini are smaller than the spaces (rsp) between the ridges (r) deposited by the worm. The length of the ridges and the distance between them is smaller than the length of anterior segments (seg). The smaller portion of the size range of tooth lengths (tl) overlaps that of the largest tooth widths (tw). The smaller tooth widths overlap in size with the gaps (g) formed by the strands (st) of the tube lining.
