# Supplementary material for: Textures and traction: how tube-dwelling polychaetes get a leg up
Source: Invertebr Biol. 2015 Mar 3;134(1):61–77. doi: 10.1111/ivb.12079 (PMC4375521; doi:10.1111/ivb.12079)
Supplement: Fig S7 — Schizobranchia insignis (Sabellidae): body and tube. A. Whole worm sans feeding crown. B. Thoracic notopodium. C. Thoracic notopodial chaetae. D. Thoracic uncinus. E. Micro-teeth typical of chaetal surfaces of the thorax. F. Longitudinal section and cut edges of tube. G. Tiny bumps typical of anterior portion of tube lining. H. Microstructure of tube lining. Size ranges for a single worm (0.9 mm diam.) indicate that segments (seg) and chaetal heads (ch) of uncini, companion chaetae (see Fig. S8), and thoracic notopodial chaetae are larger than the size of spaces (sp) and bumps (bp) associated with the inner tube. The surfaces of all chaetae examined were adorned with micro-teeth with tooth lengths (tl) and widths (tw) that overlapped the size of gaps (g) formed by the strands (st) of the tube lining. Arrangement and morphology is very similar to Eudistylia vancouveri, Fig. S8. [file ivb0134-0061-sd7.pdf]

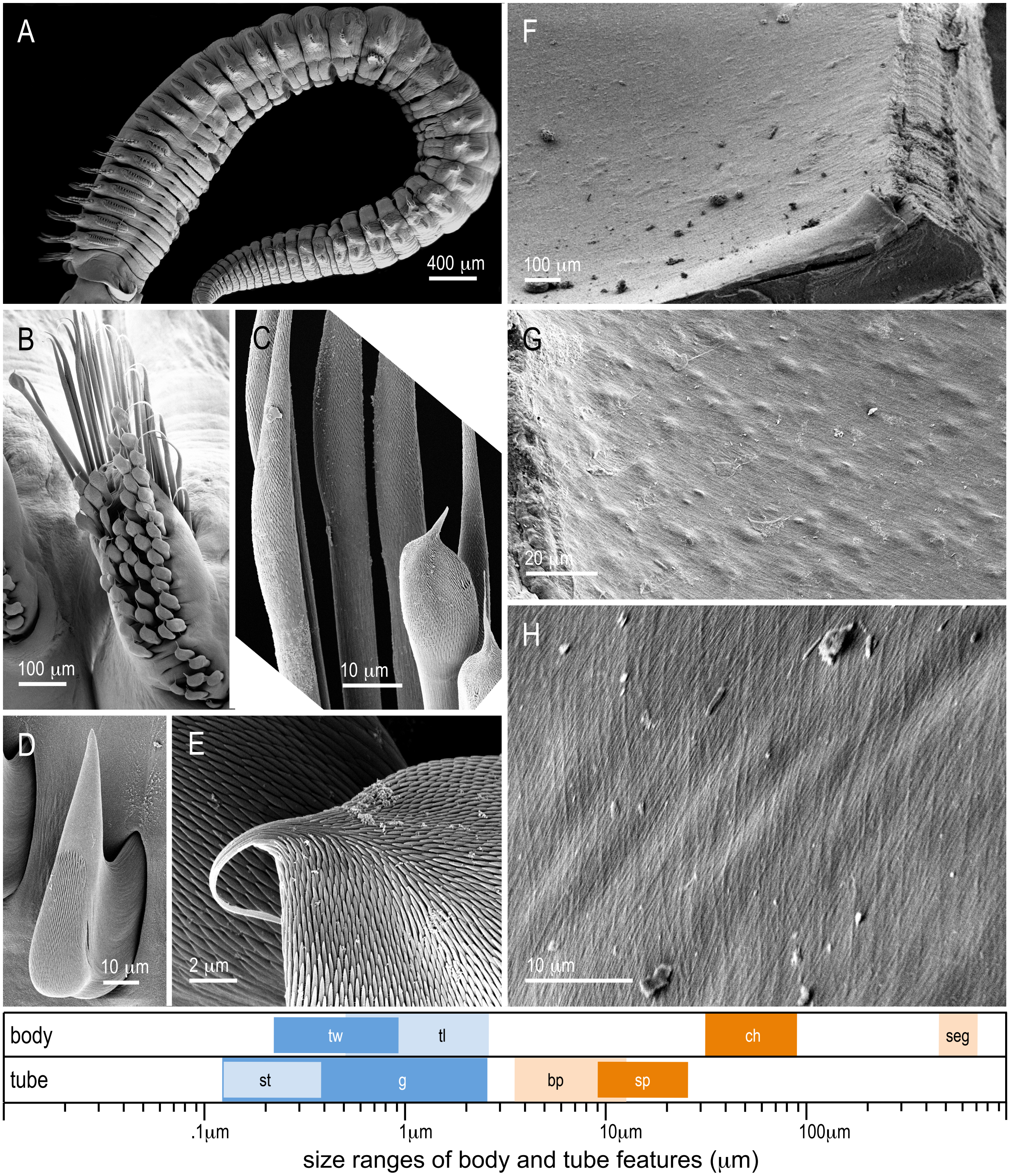

Fig. S7. *Schizobranchia insignis* (Sabellidae): body and tube. **A.** Whole worm *sans* feeding crown. **B.** Thoracic notopodium. **C.** Thoracic notopodial chaetae. **D.** Thoracic uncinus. **E.** Microteeth typical of chaetal surfaces of the thorax. **F.** Longitudinal section and cut edges of tube. **G.** Tiny bumps typical of anterior portion of tube lining. **H.** Microstructure of tube lining. Size ranges for a single worm (0.9 mm diam.) indicate that segments (seg) and chaetal heads (ch) of uncini, companion chaetae (see Fig. 8) and thoracic notopodial chaetae are larger than the size of spaces (sp) and bumps (bp) associated with the inner tube. The surfaces of all chaetae examined were adorned with microteeth with tooth lengths (tl) and widths (tw) that overlapped the size of gaps (g) formed by the strands (st) of the tube lining. Arrangement and morphology is very similar to *Eudistylia vancouveri*, see Fig. S8.
