# Supplementary material for: Textures and traction: how tube-dwelling polychaetes get a leg up
Source: Invertebr Biol. 2015 Mar 3;134(1):61–77. doi: 10.1111/ivb.12079 (PMC4375521; doi:10.1111/ivb.12079)
Supplement: Fig S8 — Eudistylia vancouveri (Sabellidae): body and tube. A. Thorax. B. Thoracic uncini and companion chaetae. C. Thoracic uncini. D. Companion chaetae. E. Tube section with smooth inner layer in contrast with rough outer surface. F. Small bumps typical of anterior inner tube lining. G. Microstructure of tube lining. The size ranges for a single worm (4.4 mm diam.) show that the lengths of the worm's segments (seg) and the chaetal heads (ch) of thoracic chaetae are larger than the tiny spaces (sp) and bumps (bp) of the inner texture. The surfaces of all chaetae were adorned with micro-teeth that had tooth widths (tw) and lengths (tl) that overlapped the size of gaps (g) formed by the strands (st) of the tube lining. Other thoracic chaetae were like those of Schizobranchia insignis (Fig. S7B–E). [file ivb0134-0061-sd8.pdf]

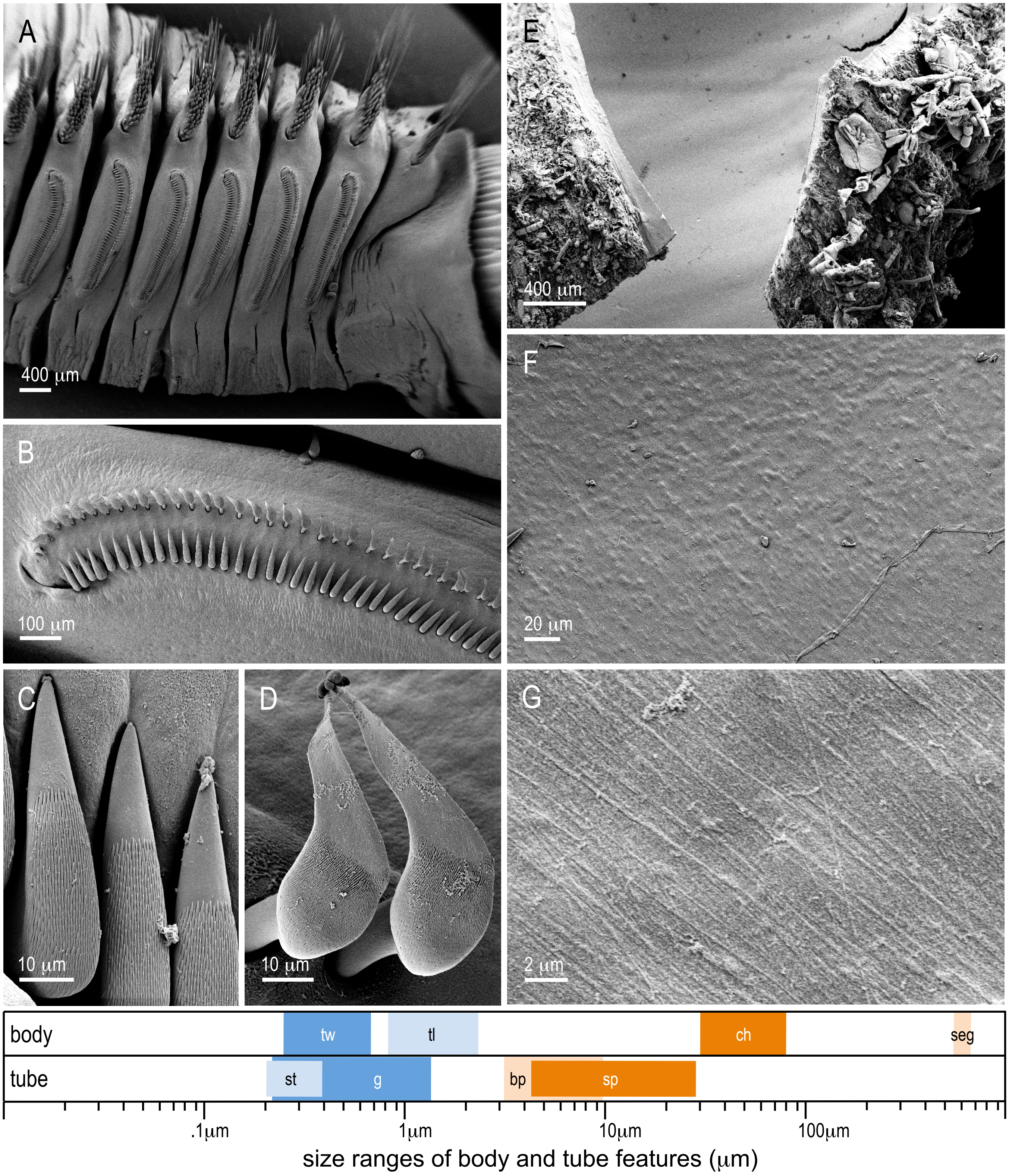

Fig. S8. *Eudistylia vancouveri* (Sabellidae): body and tube. **A.** Thorax. **B.** Thoracic uncini and companion chaetae. **C.** Thoracic uncini. **D.** Companion chaetae. **E.** Tube section with smooth inner layer in contrast with rough outer surface. **F.** Small bumps typical of anterior inner tube lining. **G.** Microstructure of tube lining. The size ranges for a single worm (4.4 mm diam.) show that the lengths of the worm's segments (seg) and the chaetal heads (ch) of thoracic chaetae are larger than the tiny spaces (sp) and bumps (bp) of the inner texture. The surfaces of all chaetae were adorned with microteeth that had tooth widths (tw) and lengths (tl) that overlapped the size of gaps (g) formed by the strands (st) of the tube lining. Other thoracic chaetae were like those of *S. insignis* (Fig. S7 B,C,D,E).
