# Supplementary material for: Textures and traction: how tube-dwelling polychaetes get a leg up
Source: Invertebr Biol. 2015 Mar 3;134(1):61–77. doi: 10.1111/ivb.12079 (PMC4375521; doi:10.1111/ivb.12079)
Supplement: Fig S9 — Pista brevibranchiata (Terebellidae): body and tube. A. Anterior segments. B. Anterior notopodium. C. Anterior uncini. D. Micro-teeth on anterior notopodial chaetae. E. Longitudinal section of tube showing internal texture provided by sediment grains incorporated into the exterior portion of the tube. F. Microstructure of tube lining with an interruption of the normally even surface. The size ranges for a single worm (2.6 mm diam.) indicate that chaetal heads (ch) of uncini are smaller than the spaces (sp) or bumps (bp) caused by sediment grains that are themselves smaller than the length of the worm's segments (seg). Chaetal dentition has a broad range of tooth lengths (tl) and widths (tw). The smaller dentition overlaps the size of gaps (g) formed by the strands (st) of the tube lining. [file ivb0134-0061-sd9.pdf]

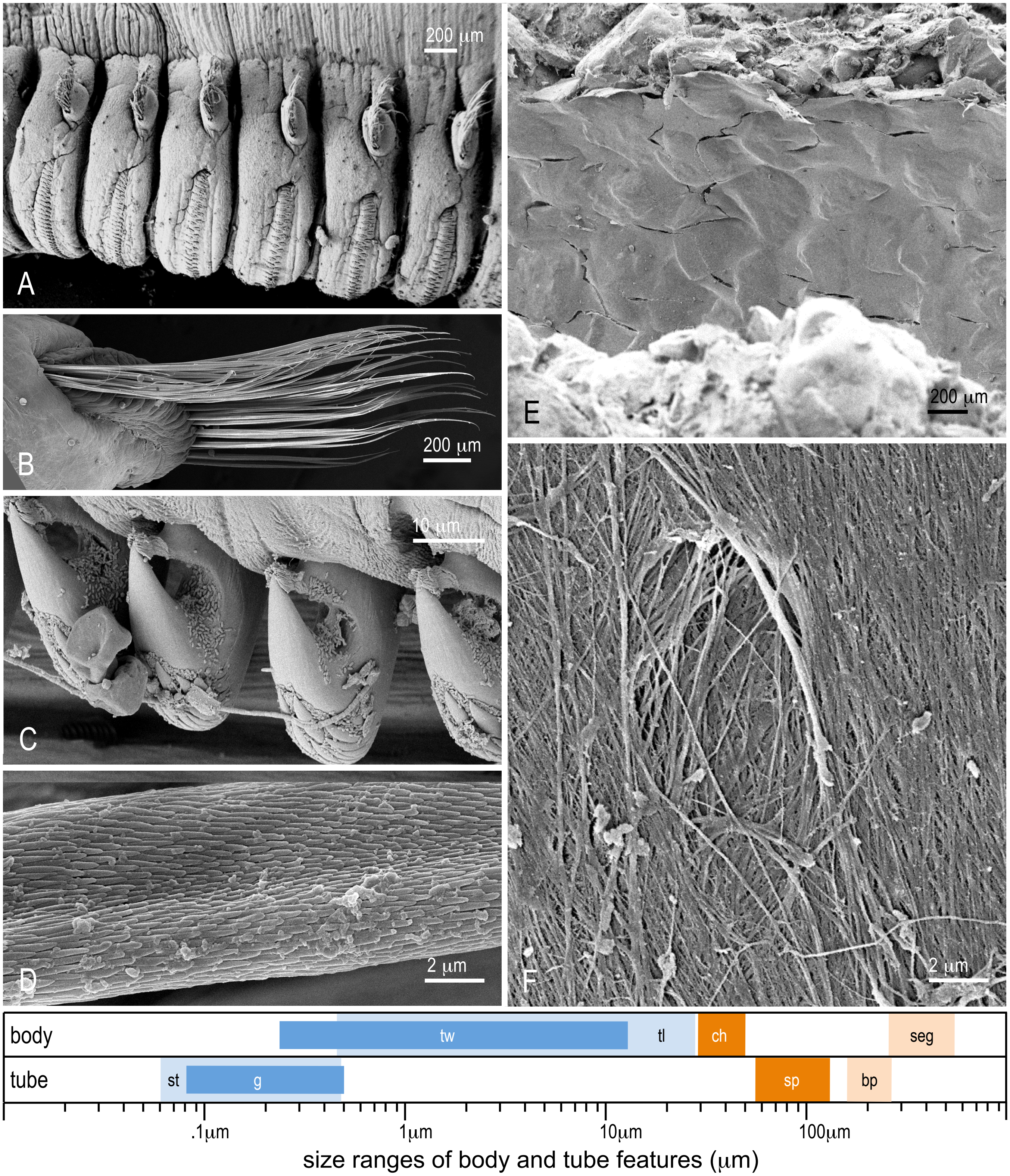

Fig. S9. *Pista brevibranchiata* (Terebellidae): body and tube. **A.** Anterior segments. **B.** Anterior notopodium. **C.** Anterior uncini. **D.** Microteeth on anterior notopodial chaetae. **E.** Longitudinal section of tube showing internal texture provided by sediment grains incorporated into the exterior portion of the tube. **F.** Microstructure of tube lining with an interruption of the normally even surface. The size ranges for a single worm (2.6 mm diam.) indicate that chaetal heads (ch) of uncini are smaller than the spaces (sp) or bumps (bp) caused by sediment grains that are themselves smaller than the length of the worm's segments (seg). Chaetal dentition has a broad range of tooth lengths (tl) and widths (tw). The smaller dentition overlaps the size of gaps (g) formed by the strands (st) of the tube lining.
