# Supplementary material for: Textures and traction: how tube-dwelling polychaetes get a leg up
Source: Invertebr Biol. 2015 Mar 3;134(1):61–77. doi: 10.1111/ivb.12079 (PMC4375521; doi:10.1111/ivb.12079)
Supplement: Fig S10 — Pectinaria gouldii (Pectinariidae): body and tube. A. Mid-body parapodium. B. Texture of capillary chaeta. C. Mid-body row of uncini. D. Uncini. E. Fractured top edge of tube. F. Fractured edge of foamy glue that attaches sand grains. G. Texture of inner tube lining including holes in the fabric perhaps caused by the worm's uncini (compare size and pattern of the dentition of the uncini and the holes). The size ranges for a single worm (5.0 mm diam.) indicate that chaetal heads (ch) of uncini are smaller than the spaces (sp) or bumps (bp) caused by sand grains which are themselves smaller that the length of the worm's segments (seg). Chaetal dentition has a broad range of tooth lengths (tl) and widths (tw). But only the smaller range of tooth widths overlaps the size of gaps (g) formed by the strands (st) of the tube lining. [file ivb0134-0061-sd10.pdf]

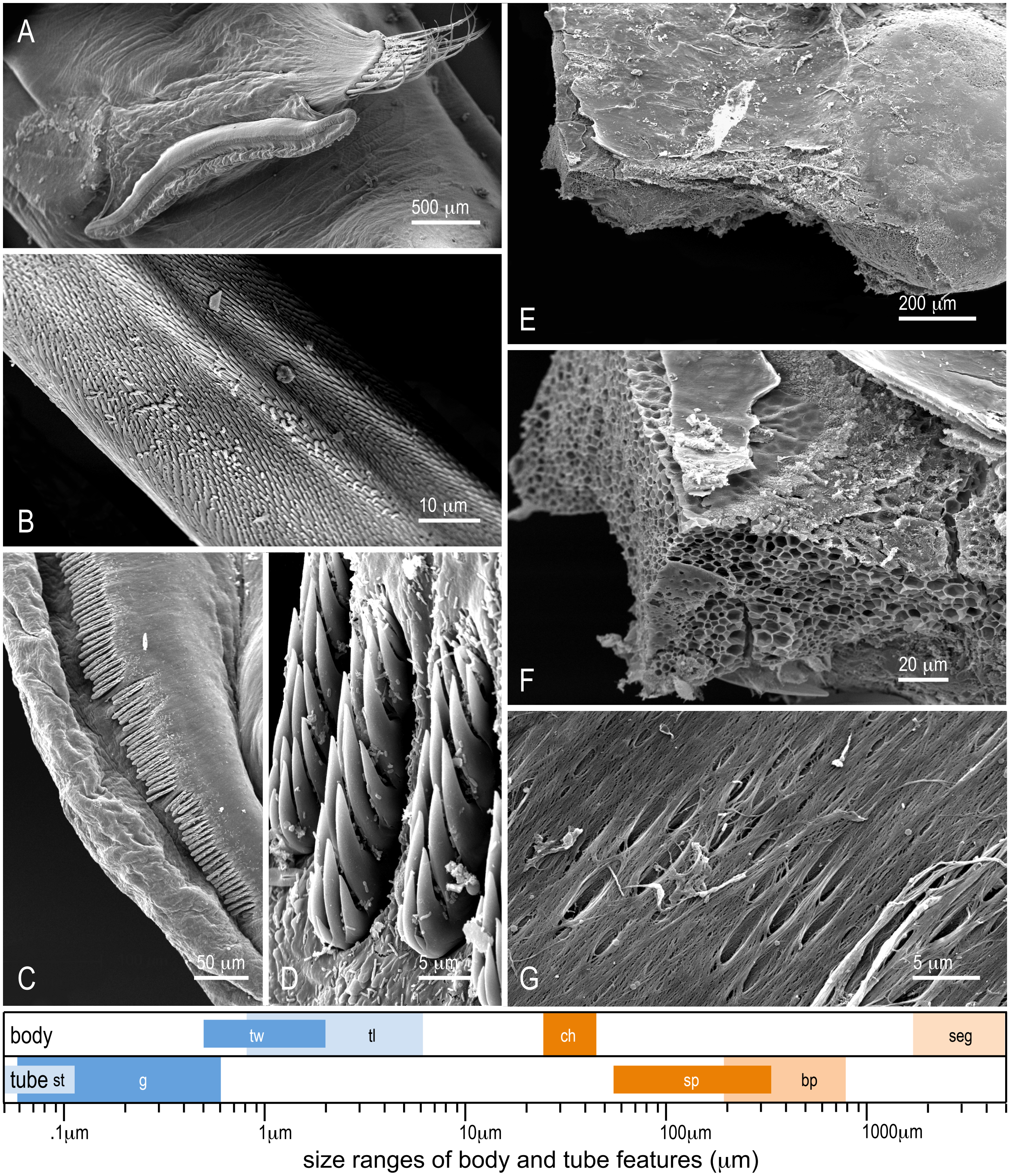

Fig. S10. *Pectinaria gouldii* (Pectinariidae): body and tube. **A.** Mid-body parapodium. **B.** Texture of capillary chaeta. **C.** Mid-body row of uncini. **D.** Uncini. **E.** Fractured top edge of tube. **F.** Fractured edge of foamy glue that attaches sand grains. **G.** Texture of inner tube lining including holes in the fabric perhaps caused by the worm's uncini (compare size and pattern of the dentition of the uncini and the holes). The size ranges for a single worm (5.0 mm diam.) indicate that chaetal heads (ch) of uncini are smaller than the spaces (sp) or bumps (bp) caused by sand grains which are themselves smaller that the length of the worm's segments (seg). Chaetal dentition has a broad range of tooth lengths (tl) and widths (tw). But only the smaller range of tooth widths overlaps the size of gaps (g) formed by the strands (st) of the tube lining.
