# Supplementary material for: Textures and traction: how tube-dwelling polychaetes get a leg up
Source: Invertebr Biol. 2015 Mar 3;134(1):61–77. doi: 10.1111/ivb.12079 (PMC4375521; doi:10.1111/ivb.12079)
Supplement: Fig S11 — Alvinella pompejana (Alvinellidae): body and tube. A. Anterior chaetal spines. B. Worn surface of spines. C. Mid-body neuropodium with uncini. D. Mid-body uncini. E. Mid-body notopodium with capillary chaetae. F. Serrated capillary chaetae. G. Inner tube lining. H. Bacteria incorporated into tube lining. The size ranges for a single worm (4.0 mm diam.) indicate that segment size (seg) falls in the middle of the range of bumps (bp) and spaces (sp) produced by incorporated material; chaetal heads (ch) overlap the smaller portion of this range. Tooth widths (tw) and lengths (tl) cover a large size range and overlap with the gaps (g) formed by the strands (st) of the tube lining. The rugosity offered by the bacteria (bact) incorporated into the tube wall is on a size scale similar to the finest dentition. [file ivb0134-0061-sd11.pdf]

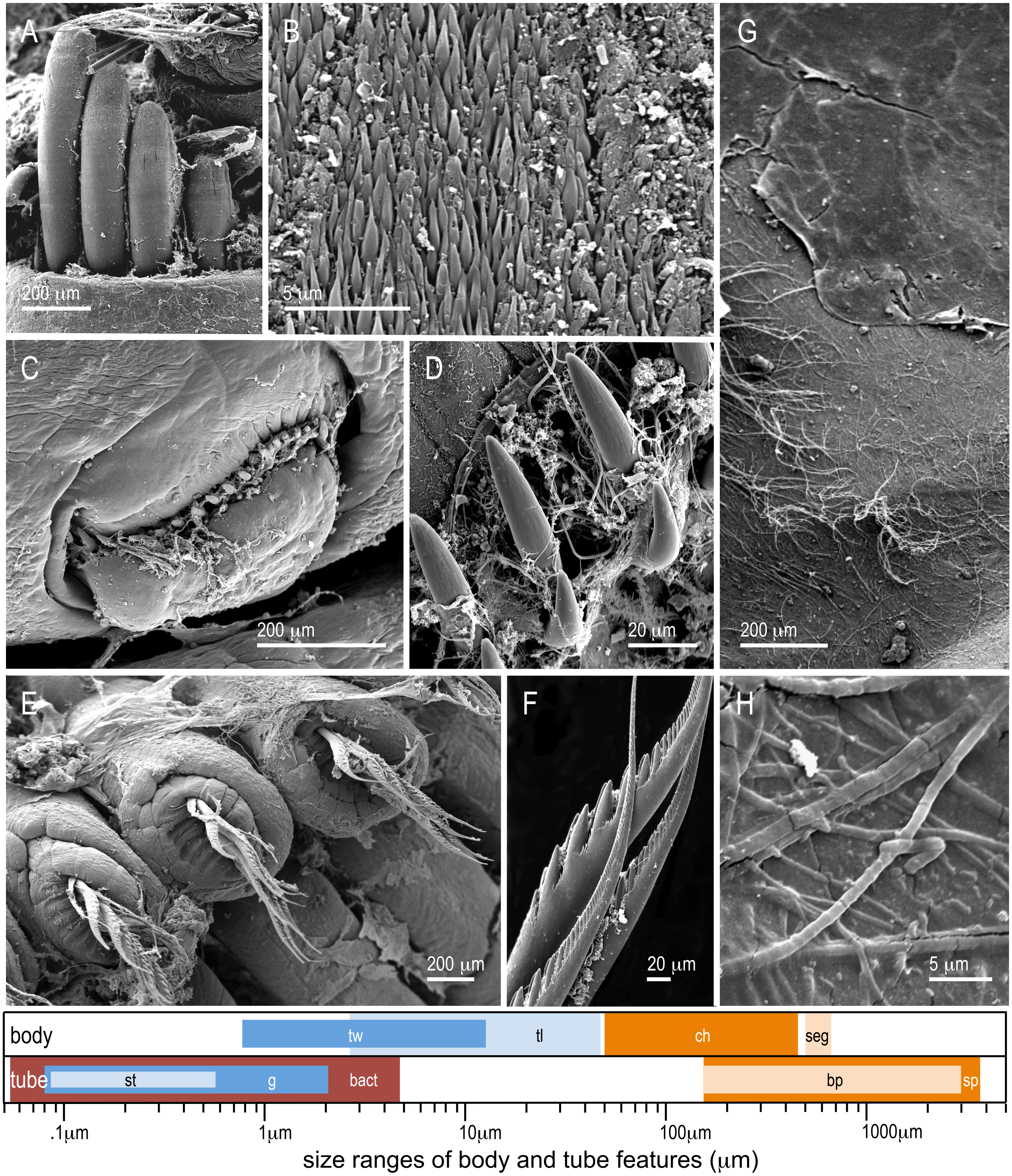

Fig. S11. *Alvinella pompejana* (Alvinellidae): body and tube. **A**. Anterior chaetal spines. **B**. Worn surface of spines. **C**. Mid-body neuropodium with uncini. **D**. Mid-body uncini. **E**. Mid-body notopodium with capillary chaetae. **F**. Serrated capillary chaetae. **G**. Inner tube lining. **H**. Bacteria incorporated into tube lining. The size ranges for a single worm (4.0 mm diam.) indicate that segment size (seg) falls in the middle of the range of bumps (bp) and spaces (sp) produced by incorporated material; chaetal heads (ch) overlap the smaller portion of this range. Tooth widths (tw) and tooth lengths (tl), cover a large size range and overlap with the gaps (g) formed by the strands (st) of the tube lining. The rugosity offered by the bacteria (bact) incorporated into the tube wall is on a size scale similar to the finest dentition.
