# Supplementary material for: Textures and traction: how tube-dwelling polychaetes get a leg up
Source: Invertebr Biol. 2015 Mar 3;134(1):61–77. doi: 10.1111/ivb.12079 (PMC4375521; doi:10.1111/ivb.12079)
Supplement: Fig S12 — Clymenella torquata (Maldanidae): body and tube. A. Posterior parapodium with capillary chaetae and row of uncini. B. Row of uncini. C. Shafts of capillary chaetae. D. Uncinus. E. Surface of capillary chaeta. F. Longitudinal section of tube. G. Tilted view of tube lining to show texture. H. Surface of lining. The size ranges of a single worm (1.8 mm diam.) show that the chaetal heads (ch) of uncini are smaller than the bumps (bp) or spaces (sp) caused by sediment incorporated into the tube's exterior. The worm's segments (seg) are much larger than these sand grains. Chaetal dentition, as represented by tooth widths (tw) and lengths (tl) of uncini and capillary chaetae, has a broad size range. The gaps (g) formed by the strands (st) of the tube lining overlap in size with the smaller portion of the range of chaetal dentition. [file ivb0134-0061-sd12.pdf]

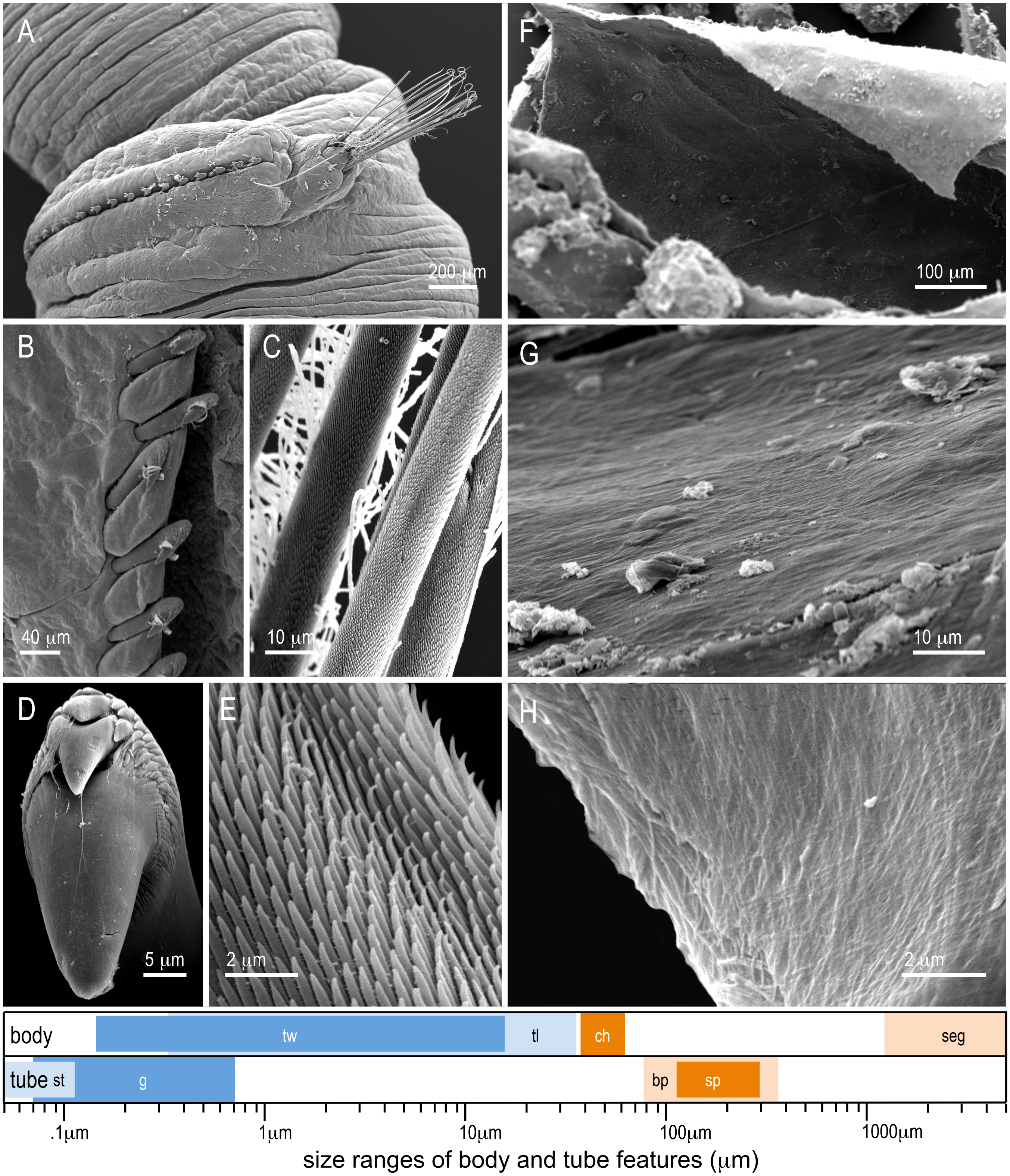

Fig. S12. *Clymenella torquata* (Maldanidae): body and tube. **A**. Posterior parapodium with capillary chaetae and row of uncini. **B**. Row of uncini. **C**. Shafts of capillary chaetae. **D**. Uncinus. **E**. Surface of capillary chaeta. **F**. Longitudinal section of tube. **G**. Tilted view of tube lining to show texture. **H**. Surface of lining. The size ranges of a single worm (1.8 mm diam.) show that the chaetal heads (ch) of uncini are smaller than the bumps (bp) or spaces (sp) caused by sediment incorporated into the tube's exterior. The worm's segments (seg) are much larger than these sand grains. Chaetal dentition, as represented by tooth widths (tw) and lengths (tl) of uncini and capillary chaetae, has a broad size range. The gaps (g) formed by the strands (st) of the tube lining overlap in size with the smaller portion of the range of chaetal dentition.
