# Supplementary material for: Textures and traction: how tube-dwelling polychaetes get a leg up
Source: Invertebr Biol. 2015 Mar 3;134(1):61–77. doi: 10.1111/ivb.12079 (PMC4375521; doi:10.1111/ivb.12079)
Supplement: Fig S13 — Diopatra ornata (Onuphidae): body and tube. A. Mid-body neuropodium. B. Hooded hook. C. Pectinate chaeta. D. Surface dentition of limbate chaeta. (B,C, and D are from mid-body neuropodia). E. Longitudinal section of tube. F. Inner tube lining. G. Wrinkled texture of inner lining. The size ranges of a single worm (4.2 mm diam.) show that the chaetal heads (ch) of hooks and pectinate chaetae are smaller than the bumps (bp) and spaces (sp) associated with materials incorporated into the tube's exterior. The worm's segments are generally larger than these features but slightly overlap the size of spaces. Tooth widths (tw) and lengths (tl) associated with the various chaetae overlap the size range of the wrinkles (wr) that form the dominant texture of the inner tube lining. The gaps (g) formed by strands (st) are smaller than the chaetal dentition. [file ivb0134-0061-sd13.pdf]

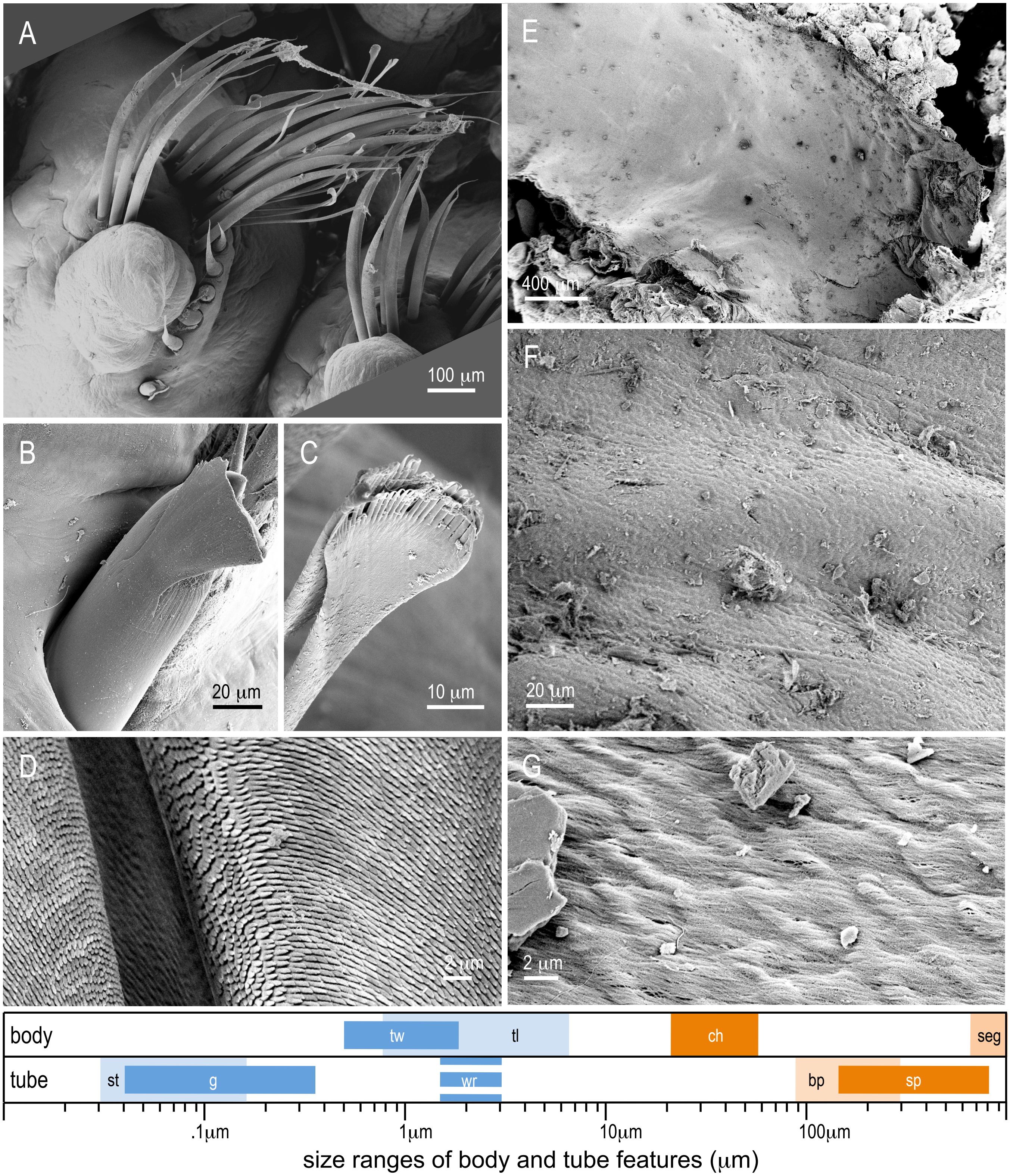

Fig. S13. *Diopatra ornata* (Onuphidae): body and tube. **A**. Mid-body neuropodium. **B**. Hooded hook. **C**. Pectinate chaeta. **D**. Surface dentition of limbate chaeta. (B,C, and D are from mid-body neuropodia). **E**. Longitudinal section of tube. **F**. Inner tube lining. **G**. Wrinkled texture of inner lining. The size ranges of a single worm (4.2 mm diam.) show that the chaetal heads (ch) of hooks and pectinate chaetae are smaller than the bumps (bp) and spaces (sp) associated with materials incorporated into the tube's exterior. The worms segments are generally larger than these features but slightly overlap the size of spaces. Tooth widths (tw) and tooth lengths (tl), associated with the various chaetae overlap the size range of the wrinkles (wr) that form the dominant texture of the inner tube lining. The gaps (g) formed by strands (st) are smaller than the chaetal dentition.
