# Supplementary material for: Textures and traction: how tube-dwelling polychaetes get a leg up
Source: Invertebr Biol. 2015 Mar 3;134(1):61–77. doi: 10.1111/ivb.12079 (PMC4375521; doi:10.1111/ivb.12079)
Supplement: Fig S14 — Platynereis bicanaliculata (Nereididae): body and tube. A. Anterior segments of worm. B. Hooks. C. Compound (jointed) falcate spinagers. D. Compound (jointed) falcigers. E. Longitudinal section of tube. F. Inner surface of tube lining. G. Texture of inner tube. The size ranges of a single worm (1.1 mm diam.) show that the chaetal heads (ch) of hooks and falcigers are smaller than the spaces (sp) and bumps (bp) formed by the inclusion of algae, organic and inorganic materials in the outer layers of the tube. The size of these materials is similar to the length of the worm's segments (seg). Strands (st) of secreted material form gaps (g) that have a large range of sizes, overlapping the size of chaetal heads and chaetal dentition characterized by tooth lengths (tl) and tooth widths (tw). [file ivb0134-0061-sd14.pdf]

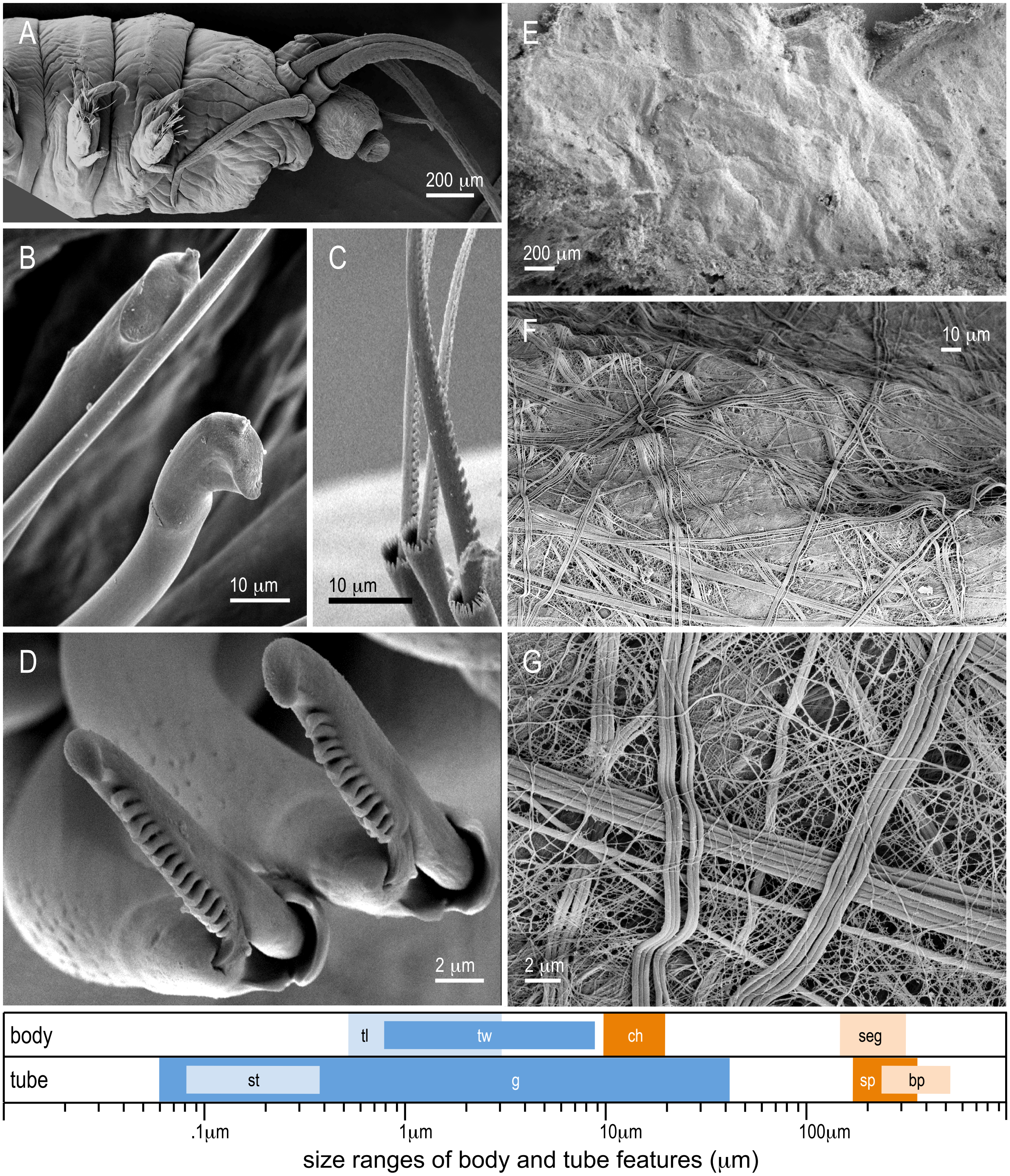

Fig. S14. *Platynereis bicanaliculata* (Nereididae): body and tube. **A**. Anterior segments of worm. **B**. Hooks. **C**. Compound (jointed) falcate spinagers. **D**. Compound (jointed) falcigers. **E**. Longitudinal section of tube. **F**. Inner surface of tube lining. **G**. Texture of inner tube. The size ranges of a single worm (1.1 mm diam.) show that the chaetal heads (ch) of hooks and falcigers are smaller than the spaces (sp) and bumps (bp) formed by the inclusion of algae, organic and inorganic materials in the outer layers of the tube. The size of these materials is similar to the length of the worm's segments (seg). Strands (st) of secreted material form gaps (g) that have a large range of sizes, overlapping the size of chaetal heads and chaetal dentition characterized by tooth lengths (tl) and tooth widths (tw).
